# Selective Photocatalytic Dehydrogenation of Formic Acid by an *In Situ*-Restructured Copper-Postmetalated Metal–Organic Framework under Visible Light

**DOI:** 10.1021/jacs.2c04905

**Published:** 2022-09-01

**Authors:** Houeida Issa Hamoud, Patrick Damacet, Dong Fan, Nisrine Assaad, Oleg I. Lebedev, Anna Krystianiak, Abdelaziz Gouda, Olivier Heintz, Marco Daturi, Guillaume Maurin, Mohamad Hmadeh, Mohamad El-Roz

**Affiliations:** † Normandie Univ, ENSICAEN, UNICAEN, CNRS, Laboratoire Catalyse et Spectrochimie, 14050 Caen, France; ‡ Department of Chemistry, American University of Beirut, P.O. Box 11-0236, Riad El-Solh, Beirut 1107 2020, Lebanon; § Normandie Univ, ENSICAEN, UNICAEN, CNRS, Laboratoire CRISMAT, UMR 6508, 14050 Caen, France; ∥ Department of Chemistry, University of Toronto, 80 St. George Street, Toronto, ON M5S 3H6, Canada; ⊥ Institut Charles Gerhardt Montpellier (ICGM), University of Montpellier, CNRS, ENSCM, 34095 Montpellier, France; # ICB, CNRS UMR 6303 − Université de Bourgogne Franche-Comté, 9 Avenue A. Savary, 21078 Dijon, France

## Abstract

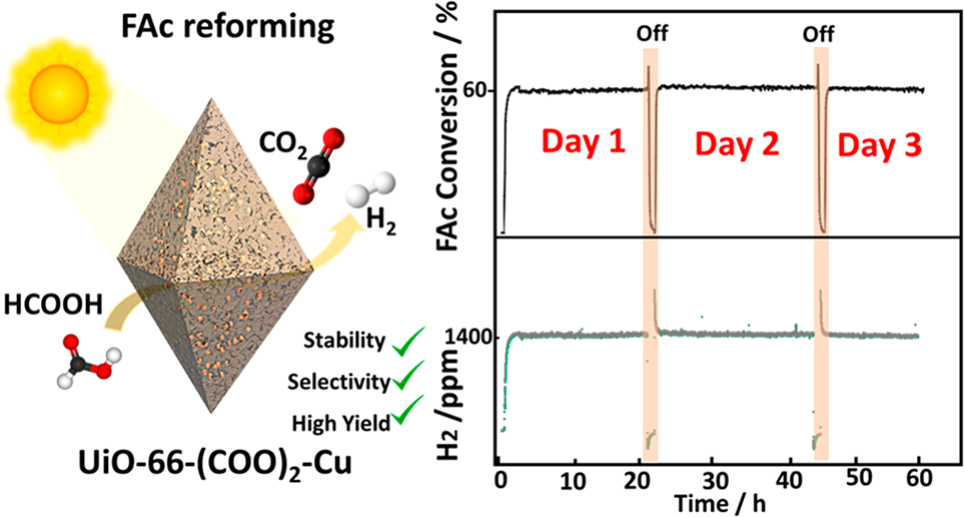

Formic acid is considered as one of the most promising liquid organic hydrogen carriers. Its catalytic dehydrogenation process generally suffers from low activity, low reaction selectivity, low stability of the catalysts, and/or the use of noble-metal-based catalysts. Herein we report a highly selective, efficient, and noble-metal-free photocatalyst for the dehydrogenation of formic acid. This catalyst, UiO-66(COOH)_2_-Cu, is built by postmetalation of a carboxylic-functionalized Zr-MOF with copper. The visible-light-driven photocatalytic dehydrogenation process through the release of hydrogen and carbon dioxide has been monitored in real-time *viaoperando* Fourier transform infrared spectroscopy, which revealed almost 100% selectivity with high stability (over 3 days) and a conversion yield exceeding 60% (around 5 mmol·g_cat_^–1^·h^–1^) under ambient conditions. These performance indicators make UiO-66(COOH)_2_-Cu among the top photocatalysts for formic acid dehydrogenation. Interestingly, the as-prepared UiO-66(COOH)_2_-Cu hetero-nanostructure was found to be moderately active under solar irradiation during an induction phase, whereupon it undergoes an *in-situ* restructuring process through intraframework cross-linking with the formation of the anhydride analogue structure UiO-66(COO)_2_-Cu and nanoclustering of highly active and stable copper sites, as evidenced by the *operando* studies coupled with steady-state isotopic transient kinetic experiments, transmission electron microscopy and X-ray photoelectron spectroscopy analyses, and Density Functional Theory calculations. Beyond revealing outstanding catalytic performance for UiO-66(COO)_2_-Cu, this work delivers an in-depth understanding of the photocatalytic reaction mechanism, which involves evolutive behavior of the postmetalated copper as well as the MOF framework over the reaction. These key findings pave the way toward the engineering of new and efficient catalysts for photocatalytic dehydrogenation of formic acid.

## Introduction

Hydrogen is one of the most basic and most abundant elements on this planet. However, it is rarely found in its molecular form, being essentially present in molecular compounds.^1^ Therefore, it needs to be extracted through different processes. Molecular hydrogen has experienced a global resurgence lately as an energy carrier and a secondary source of green energy.^2^ Despite being characterized by a low atomic weight, low boiling point, high flammability range, and extremely low density, this species possesses an enormous gravimetric energy (chemical energy per mass unit) of 120 kJ·g^–1^, which surpasses those of methane, gasoline, and ethanol (56, 47, and 30 kJ·g^–1^, respectively).^3^ Therefore, the development of cheap, safe, and effective hydrogen on-site catalytic production systems has been gaining much attention in the past few years.^4,5^ Along with the various thermal, photocatalytic, electrical, and biological processes utilized, including fermentation, biophotolysis, alkaline electrolysis, and water-splitting-based solar energy, steam reforming processes are considered the most feasible route for hydrogen production.^6^ In such processes, hydrogen is generated *via* catalyzed endothermic equilibrium reactions between a hydrocarbon (*e.g.*, methanol, acetone, methane, formic acid, or ethylene glycol) and steam.^7^

Recently, formic acid (HCOOH) has been attracting much attention as a promising hydrogen source and liquid organic hydrogen carrier species.^8−10^ This is due to its advantageous properties including its low toxicity, nonflammability, high stability, biodegradability, and availability, as more than 600,000 megatons of formic acid are produced worldwide on a yearly basis.^4,11^ In this regard, formic acid is recognized as one of the most promising hydrogen carriers, with a H_2_ volumetric capacity of 53 kg·m^–3^, which corresponds to a huge energy density of 1.76 kW·h·L^–1^.^12^ What makes formic acid an excellent H_2_ storage and production system is the fact that hydrogen release occurs spontaneously under mild conditions, resulting in an exergonic process,^13−15^ as opposed to other carriers such as methanol, acetic acid, acetaldehyde, and ammonia, which require temperatures above 373 K to release H_2_ and have other drawbacks such as poor selectivity, where more than one major product can be obtained.^16−18^

Formic acid, which contains approximately 4.4 wt % hydrogen, degrades in the absence of catalyst *via* two low-enthalpy routes associated with the following chemical reactions:^9^dehydrogenation (decarboxylation):
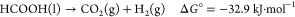
1dehydration (decarbonylation):
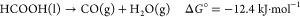
2The dehydrogenation of formic acid is a desired and indispensable pathway for all systems using this chemical species as a hydrogen storage and production medium. Although this route involves the generation of CO_2_ along with H_2_, the former can be thereafter hydrogenated using any appropriate catalyst, resulting in the regeneration of formic acid *via* a carbon-free emission cycle.^19,20^ On the other hand, the second route illustrated by the dehydration of formic acid is assumed to be undesired because the generation of carbon monoxide results in poisoning of the hydrogen cell’s catalyst, leading to a lower overall hydrogen yield.^21^

A tremendous amount of research has been conducted during the past few years on the development of convenient homogeneous and heterogeneous catalysts for formic acid dehydrogenation.^22−24^ For instance, after Coffey initially reported the first homogeneous catalytic system based on Ru, Ir, and Pt phosphine complexes for the dehydrogenation of formic acid in 1967,^25^ various research groups have been involved in the development and investigation of new active homogeneous catalysts based on noble metals, bipyridine moieties, and pincer-type ligands.^26^ The heterogeneous systems are mostly made of metal oxides and supported metallic nanoparticles, and noble-metal-based catalysts are known to be the most efficient and sustainable ones in the latter category.^27−29^ Although acceptable catalytic activities have been achieved using heterogeneous catalysts, their poor stability under acidic conditions was a major concern.^30,31^ Moreover, most metal oxides employed as photo- or thermoactive catalysts, including TiO_2_ nanoparticles, resulted in low sustainability, low selectivity toward H_2_, and the formation of CO as a side product under mild conditions.^32,33^

Metal–organic frameworks (MOFs) are a new and promising class of crystalline porous hybrid materials composed of metal ion clusters linked by organic linkers *via* strong covalent bonds to form extended networks.^34−36^ Due to their outstanding features, including high internal surface area, large porosity, structural tunability, potentially high density of active sites, and acceptable thermal and chemical stability,^37,38^ MOFs have attracted much attention in the field of heterogeneous catalysis.^39−48^ They have been used as effective supports to immobilize metal nanoparticles in their pores and on the functional groups within the backbone of the framework, which prevents aggregation of the nanoparticles.^49^

There have been very few reports on the decomposition of formic acid into hydrogen and carbon dioxide using MOFs as catalysts to date. In particular, the incorporation of palladium and palladium–gold nanoparticles was achieved in two different MOF frameworks, MIL-125 and MIL-101, respectively.^49,50^ Although good formic acid conversion was achieved over these MOF-based catalysts, their stability and selectivity were much lower than those of the existing homogeneous catalysts.^14,51^ Nevertheless, a high selectivity toward H_2_ was achieved using a ruthenium complex immobilized on a newly synthesized phosphine-functionalized MOF known as LSK-15. However, a moderate conversion rate was obtained at temperatures higher than 125 °C.^52^

Herein we present the visible-light-driven dehydrogenation of formic acid into H_2_ over a copper-postmetalated zirconium MOF, namely, UiO-66-(COOH)_2_-Cu. Remarkably, this engineered photocatalyst was demonstrated to have high selectivity (>99.9%) and ultrahigh stability and to give formic acid conversions of 60% at room temperature and more than 90% at 150 °C, which were achieved for three continuous days without loss of efficiency and selectivity. The formic acid photoconversion and the hydrogen and carbon dioxide evolutions were monitored in real time using *operando* Fourier transform infrared (FTIR) spectroscopy, where isotopically enriched H^12^COOH/H^13^COOH/D^12^COOH reactants were employed to characterize the photoactivity and selectivity of the catalyst and, in tandem with density functional theory (DFT) calculations, to elucidate the catalytic reaction mechanism, which involves an uncommon evolutive behavior of both the postmetalated Cu species and the MOF framework. Finally, the temperature dependence of the kinetics and thermodynamics of the catalytic reaction was systematically examined to assess the promise of the catalyst for further applications.

## Experimental Section

### Materials

All chemical reagents and solvents mentioned in this work were commercially supplied and used directly without any additional purification. Zirconyl chloride octahydrate (ZrOCl_2_·8H_2_O), 1,2,4,5-benzenetetracarboxylic acid, methanol (gradient grade, 99.93%), dimethylformamide (DMF) (ACS grade), and copper(II) nitrate were purchased from Sigma-Aldrich.

### Synthesis of UiO-66(COOH)_2_

UiO-66(COOH)_2_ was synthesized under conditions similar to those reported in the literature.^53^ In brief, equimolar amounts of zirconyl chloride octahydrate (59.4 mg, 0.184 mmol) and 1,2,4,5-benzenetetracarboxylic acid (47.1 mg, 0.184 mmol) were dissolved in 4 mL of DMF that had been placed in a 20 mL scintillation vial, and the mixture was sonicated for a couple of minutes. Then 4 mL of formic acid modulator was later added to the obtained mixture, followed by sonication for a few extra minutes. The reaction mixture was then placed in a preheated oven at 130 °C for 5 h. The obtained white powder was collected by centrifugation at 4000 rpm and washed five times with DMF and three times with MeOH. UiO-66(COOH)_2_ was then dried under dynamic vacuum oven at 110 °C overnight.

### Preparation of UiO-66(COOH)_2_-Cu

In a 20 mL scintillation vial, 30 mg of copper nitrate was dissolved in 15 mL of DMF by sonication for 10 min until a clear solution was obtained. Then 60 mg of UiO-66(COOH)_2_ was added to the copper solution, followed by sonication for few minutes to ensure full MOF dispersion in the solution. The mixture was then stirred on a hot plate at 75 °C for 21 h until a brownish microcrystalline powder was obtained. The supernatant was discarded by centrifugation, and the solids were washed with DMF for 2 days, with fresh DMF being exchanged three times per day followed by fresh methanol for another 2 days. Finally, the solids were collected by centrifugation and dried in a vacuum oven at 80 °C overnight.

### Characterization

Powder X-ray diffraction (PXRD) analyses of UiO-66-(COOH)_2_ and UiO-66-(COOH)_2_-Cu were carried out using a Bruker D8 Advance X-ray diffractometer (Bruker AXS GmbH, Karlsruhe, Germany) with Cu Kα radiation (λ = 1.5418 Å), a voltage of 40 kV, a current of 40 mA, and a 2θ range from 5° to 50° with an increment of 0.02°. Nitrogen adsorption/desorption measurements were performed with an ASAP 2020 MP instrument. The specific surface area was calculated with the Brunauer–Emmett–Teller (BET) equation, while the pore volumes were determined by the Barrett–Joyner–Halenda (BJH) method. Prior to the measurements, samples were activated under dynamic vacuum at 110 °C for 6 h. The content and distribution of Cu and Zr were determined by scanning electron microscopy–energy dispersive X-ray spectroscopy (SEM-EDX) on a JEOL JSM-5500LV microscope or a MIRA TESCAN microscope. The images were collected with an acceleration voltage of 30 kV. The amount of copper incorporated in the framework was determined using an iCE 3000 series atomic absorption spectrophotometer after digestion of the catalyst in aqua regia and hydrofluoric acid (HF) solutions (more details are given in the SI).

X-ray photoelectron spectroscopy (XPS) measurements were carried out on an Versaprobe electron spectrometer (ULVAC-PHI) with a base vacuum in the analysis chamber on the order of 10^–8^ Pa. The samples were irradiated with monochromatized Al Kα radiation with a photon energy of 1486.6 eV. The resolution measured by the FWHM of the Ag 3d_5/2_ line was 0.6 eV for the setting used during acquisitions. Energy calibration was performed on the C 1s line of adventitious carbon at 284.8 eV. CasaXPS was used for data treatment. Advanced transmission electron microscopy (TEM) was carried out on an aberration-, probe-, and image-corrected JEM ARM200F cold field-emission gun microscope operated at 200 kV equipped with a CENTURIO EDX detector and GIF Quantum spectrometer. Diffuse-reflectance UV–vis measurements relevant to the speciation and oxidation state of Cu were carried out using a Cary 4000 UV–vis spectrophotometer and a Harrick Praying Mantis diffuse reflectance accessory. All of the spectra were recorded between 200 and 800 nm using an average time of 0.2 s and a scan rate of 300 nm·min^–1^.

Nanosecond transient absorption experiments were performed using a commercial transient absorption spectrometer (Edinburgh Instruments LP 980) pumped with a nanosecond 10 Hz Nd:YAG laser and harmonic crystals (266 nm; 355 and 532 nm). The samples were analyzed at a rate of 1 Hz and prepared in a 1 cm × 1 cm quartz cuvette to an absorbance value of around 0.2 OD of dispersed MOF (around 1 mg) in dry CH_3_CN.

### Photocatalytic Tests

For the *operando* experiments, a “Sandwich” IR cell reactor (Scheme S1) was used to study the performance of UiO-66-(COOH)_2_ and its Cu-metalated derivative during the photodecomposition of formic acid (FAc) under visible-light irradiation at room temperature (RT = 25 °C). The catalyst, as a self-supported pellet with a mass of about 20 mg, was first activated in Ar at RT under visible-light irradiation using a Xe lamp (Hamamatsu LC8, irradiance = 71 mW/cm^2^) with a >390 nm high-pass filter. Then the reaction was studied in the presence of 2400 ppm FAc at a total flow rate of 25 cm^3^·min^–1^ in argon. Additional tests were performed in the presence of H^13^COOH and DCOOH. The relative concentrations of the effluent gas were stabilized before being sent to the cell, and then adsorption of FAc on the catalyst surface was performed in the dark before the lamp was turned on. Finally, the composition of the output gas from the IR reactor cell was analyzed simultaneously by mass spectrometry on a Pfeiffer Omnistar GSD 301 quadrupole mass spectrometer and IR spectroscopy on a ThermoNicolet NEXUS 670 FTIR spectrometer equipped with an MCT detector with a spectral resolution of 4 cm^–1^ and accumulating 64 scans. The concentration of FAc in the gas phase was calculated using the surface area of the IR band at 1109–1101 cm^–1^ and the formic acid MS signals (*m*/*z* 45 and 46). The CO_2_ and CO selectivities were determined using the IR band areas at 2395–2182 and 2140–2020 cm^–1^, respectively. The amounts of hydrogen were determined by its MS signal at *m*/*z* 2 after correction for water contributions. The FAc conversion (expressed in percent or millimoles per gram of photocatalyst per irradiated surface) and the selectivity were calculated at the steady-state using the calibration curves for different products of the reaction. It should be noted that the irradiated surface area of the pellet was about 1.6 cm^2^ (∼20% of the total surface area (2 cm^2^) was not irradiated due to the metallic holder shadow effect).

### Computational Methods

To account for the formation of the anhydride form of the Cu-metalated MOF evidenced experimentally upon light irradiation and formic acid adsorption, we adopted our previously constructed anhydride model for UiO-66-(COOH)_2_, labeled as UiO-66-(COO)_2_, with an anhydride bridge formed between adjacent ligands.^54^ All of the calculations for the reaction process were further realized on a representative cluster model of UiO-66-(COO)_2_ loaded with a single Cu as model system, as shown in Figure S1 and denoted thereafter as UiO-66-(COO)_2_-Cu. All of the DFT computations were performed using the Vienna *Ab Initio* Simulation Package (VASP), version 5.4.4,^55^ with the projector augmented wave (PAW) method to describe the pseudopotential. The electron exchange–correlation functional was treated by the Perdew–Burke–Ernzerhof functional within the generalized gradient approximation (GGA) scheme.^56^ The energy cutoff of the plane waves was set to 520 eV with an energy (force) precision of 10^–5^ eV (0.01 eV·Å^–1^). The van der Waals interactions were also included by using Grimme’s DFT-D3 method.^57^ The Brillouin zone was sampled with a 1 × 1 × 1 Monkhorst–Pack **k**-point grid for geometry optimization. The transition state of the reaction was confirmed using the climbing-image nudged elastic band (CI-NEB) approach.^58^ To evaluate the adsorption strengths of intermediates and the catalytic performance of each elementary step, the Gibbs free energy change (Δ*G*) relative to the total free energy of UiO-66-(COO)_2_-Cu and gas-phase HCOOH was calculated using eq 3:
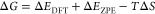
3where Δ*E*_DFT_, Δ*E*_ZPE_, *T*, and Δ*S* are the DFT-calculated electronic energy, the zero-point energy, the environment temperature (298.15 K), and the entropy, respectively.

## Results and Discussion

### MOF Catalyst Synthesis and Characterization

**Figure 1:**
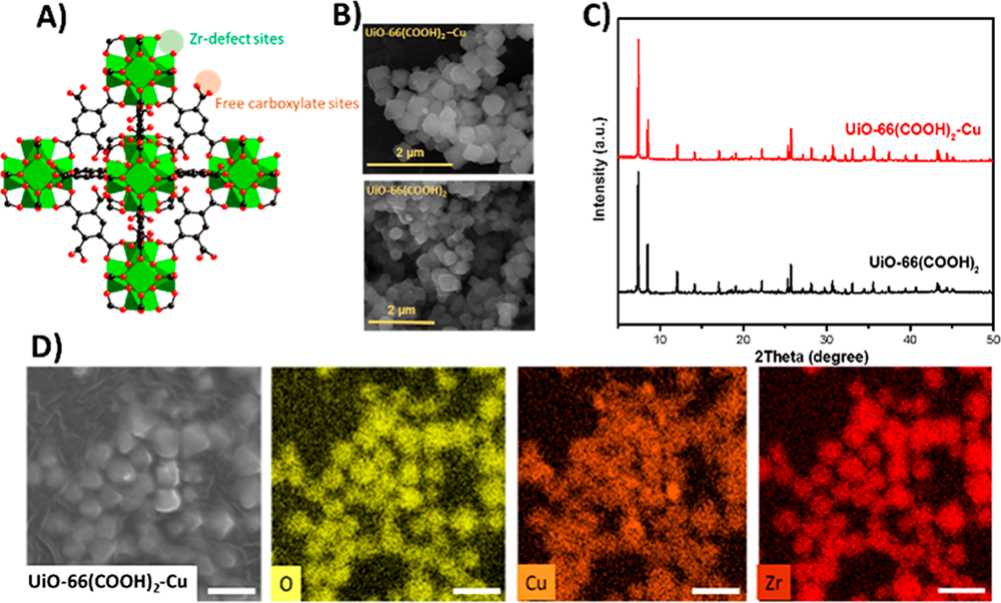
(A) Structure of UiO-66-(COOH)_2_ showing the possible anchoring sites for Cu (Zr open metal sites created by missing linkers and free carboxylic functions). (B) SEM images of UiO-66-(COOH)_2_ crystals before and after copper metalation. (C) PXRD patterns of UiO-66-(COOH)_2_ and its metalated form. (D) EDX mapping of UiO-66-(COOH)_2_-Cu showing the distributions of Cu and Zr in the MOF crystals.

UiO-66-(COOH)_2_ MOF crystals were solvothermally synthesized using formic acid as the modulator according to a previously published procedure.^53^ For the metalation, copper nitrate was employed as the metal source, which was added to the UiO-66-(COOH)_2_ crystals dispersed in DMF at 75 °C overnight. Atomic absorption spectroscopy (AAS) was used to quantify the total amount of copper incorporated in the framework, which was found to be 18 wt%. Scanning electron microscopy (SEM) images and energy-dispersive X-ray (EDX) mapping analyses further confirmed the successful metalation of UiO-66-(COOH)_2_ and revealed a homogeneous distribution of metal sites and the coexistence of Zr and Cu in all crystals (Figure 1). The obtained ratio from EDX analysis was also in agreement with the AAS results. Powder X-ray diffraction (PXRD) patterns were recorded before and after metalation and demonstrated the phase purity and the high crystallinity of UiO-66-(COOH)_2_ (Figure 1). Furthermore, no additional peaks related to crystalline copper species were observed, which confirmed that copper was anchored to the framework in its cationic form. The oxidation state and the form of the copper species will be discussed further in the photocatalytic reaction section. In order to assess the porosity of the synthesized MOF catalyst, N_2_ isotherms were measured on UiO-66-(COOH)_2_ and its Cu-metalated derivative (Figure S2). The BET surface areas were measured to be 240 and 48 m^2^·g^–1^ for UiO-66-(COOH)_2_ and UiO-66-(COOH)_2_-Cu, respectively. This decrease in the BET surface area is the result of the postmetalation process. A decrease in the pore volume was observed as well, with UiO-66-(COOH)_2_ showing a pore volume of 0.16 cm^3^·g^–1^ compared to 0.05 cm^3^·g^–1^ for the copper-postmetalated MOF.

### Activity, Selectivity, and Stability of the UiO-66-Based Photocatalysts

**Figure 2:**
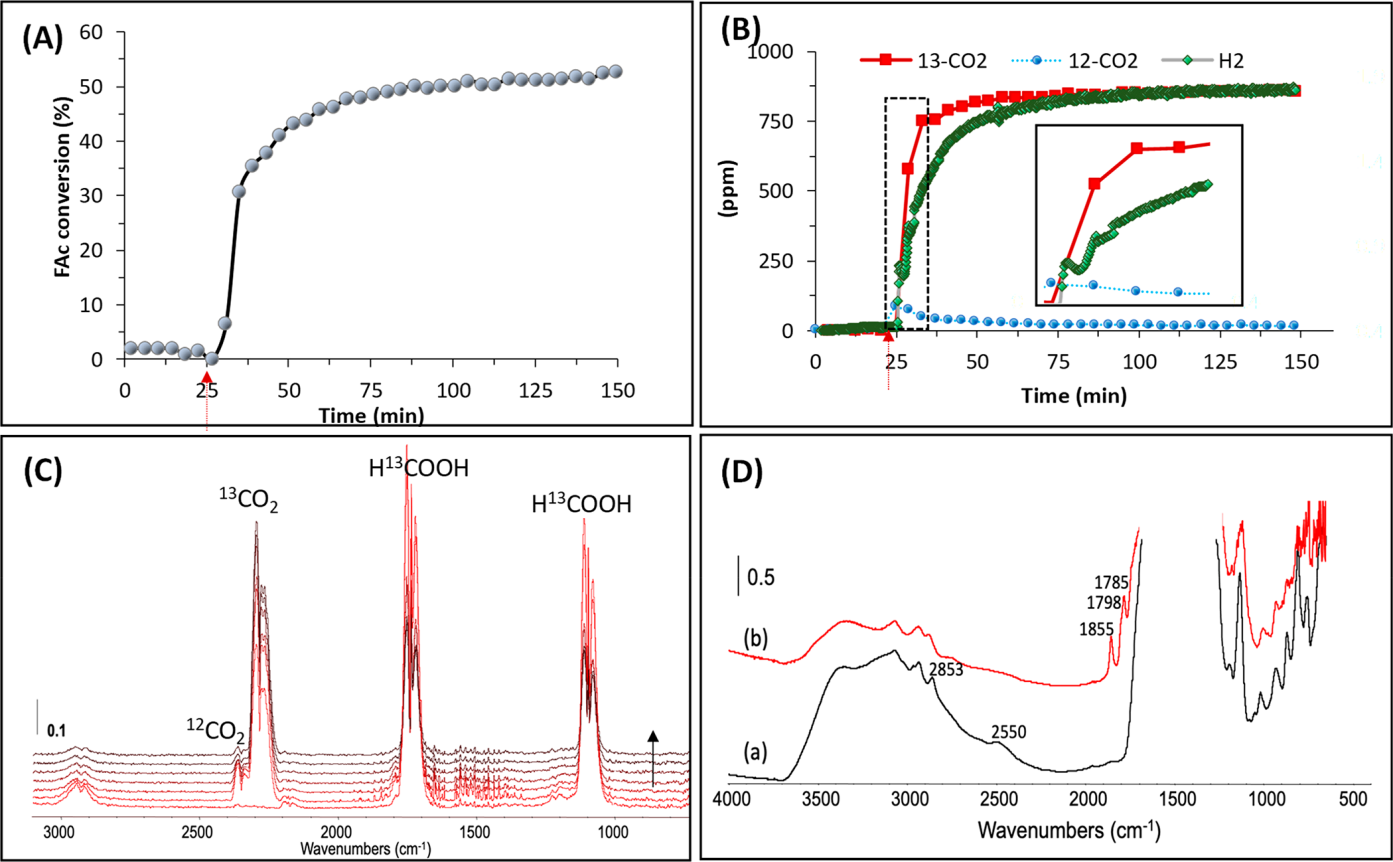
Evolution of (A) the formic acid conversion, (B) the ^13^CO_2_, ^12^CO_2_, and H_2_ quantities during HCOOH reforming (inset: zoomed-in view on the first minutes of reaction; the arrow shows the light-on time), and (C) the FTIR spectra of the reaction gas phase during the first minutes (4 min/spectrum) of the reaction. (D) FTIR spectra of the UiO-66-(COOH)_2_-Cu sample at steady state (a) in the dark and (b) during the reforming of HCOOH (labeled with ^13^C at 99% (H_2_O < 5%)) under visible-light irradiation. Reaction conditions: total flow rate = 25 cm^3^·min^–1^; [HCOOH-^13^C] = 2400 ppm (0.24%) in Ar; *T* = 25 °C; 150 W Xe lamp with a visible-light-pass filter (λ > 390 nm); irradiance = 71 mW·cm^–2^; *m*_cat_ = 20 mg (self-supported pellet with a surface area of 1.6 cm^2^).

The photocatalytic performance of UiO-66-(COOH)_2_-Cu for the dehydrogenation of formic acid was tested under flow conditions (25 cm^3^·min^–1^ with 0.25% of HCOOH in Ar) and under visible-light irradiation using an *operando* IR reactor^59^ (Scheme S1). This reactor allows simultaneous and real-time investigation of the modification on the photocatalyst surface as well as the reaction gas phase. The amounts of CO_2_ and H_2_ produced from this reaction were analyzed by gas FTIR spectroscopy and/or mass spectrometry, respectively. In order to verify the origin of the produced CO_2_, ^13^C-labeled formic acid (H^13^COOH) was used as the reactant. The evolution of the formic acid conversion over time demonstrates an increase in the first minutes of irradiation before a steady state is reached after 25 min (Figure 2A). A similar trend was observed for ^13^CO_2_, with traces of ^12^CO_2_ (5 to ∼1% at steady state) detected in the first minutes of irradiation (Figure 2B,C). The latter probably originated from the residual formic acid used during the UiO-66-(COOH)_2_ synthesis in addition to the H^12^COOH impurity (1%) already present in the H^13^COOH sample. The selective production of ^13^CO_2_ at steady state demonstrates the photocatalytic dehydrogenation of the ^13^C-labeled formic acid and the photochemical stability of the UiO-66-(COOH)_2_-Cu structure during the reaction under the operating conditions.

Decisively and based on the carbon balance, the selectivity of the formic acid dehydrogenation in this reaction was found to be more than 99%, where no CO was detected in the gas phase of the reaction. This outcome was also confirmed by the equimolar production of H_2_ and CO_2_ at the steady state. Remarkably, during the first hundred minutes of the reaction, the H_2_ evolution follows a different profile than that of formic acid conversion and CO_2_ production. This trend is unusual for a pure photocatalytic dehydrogenation process, where each molecule of CO_2_ should be accompanied by a molecule of H_2_ (Figure 2B,C). The experiment was repeated four times, and this behavior was completely reproducible. This observation suggests a possible interaction between FAc and the MOF structure at the beginning of the reaction under visible-light irradiation. IR analysis of the photocatalyst surface demonstrates the appearance of new bands at around 1855, 1798, and 1785 cm^–1^ in the first minutes of the photocatalytic reaction (Figure 2D). These bands are characteristic of anhydride functions, which were previously observed during the thermal activation of UiO-66-(COOH)_2_.^54^ Indeed, Clet *et al.* attributed these bands to thermal dehydration of the free carboxylic groups of the MOF structure, resulting in the formation of the bridged anhydrides at relatively high temperature (>100 °C). The resulting structure is labeled as UiO-66-(COO)_2_-Cu. For comparison, the IR spectra of the sample before and after 20 min of visible-light activation at 25 °C under pure Ar carrier gas (FAc-Free) demonstrated only complete removal of the adsorbed water from the surface of the catalyst without detection of the characteristic anhydride bands. In addition, only the bands of adsorbed HCOOH were detected at the steady state after HCOOH adsorption (Figure S3). These reference tests explain the delay between the CO_2_ and H_2_ production, where the protons of formic acid could be involved in the restructuring of UiO-66-(COOH)_2_-Cu to UiO-66-(COO)_2_-Cu.

**Figure 3:**
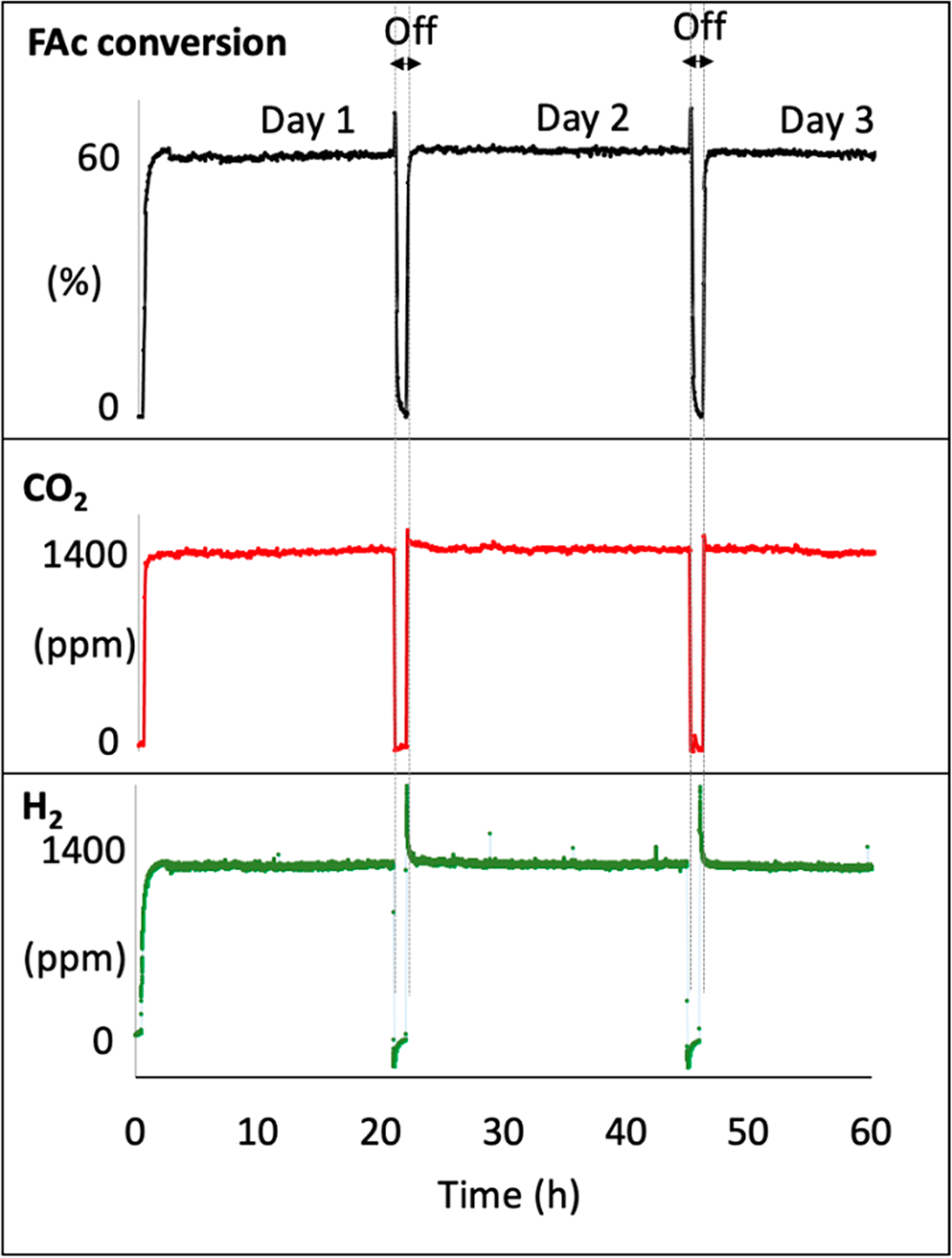
Evolution of the HCOOH conversion and the corresponding gas-phase products during three cycles/days. The regions designated by arrows correspond to the dark stages. Reaction conditions: total flow rate = 25 cm^3^·min^–1^; [HCOOH-^13^C] = 2400 ppm (0.24%) in Ar; *T* = 25 °C; 150 W Xe lamp with a visible-light-pass filter (λ > 390 nm); irradiance = 71 mW·cm^–2^; *m*_cat_ = 20 mg (self-supported pellet with a surface area of 1.6 cm^2^).

Before the in-depth characterization of this restructuring phenomena, the stability of the photocatalyst was tested in three photocatalytic cycles for around 24 h each (Figure 3). The results demonstrate ultrahigh stability of the sample during the reaction for three successive days without any significant deactivation while maintaining a selectivity of 100% (no detection of CO; Figure 2C). Moreover, the structural and chemical stability of the samples were confirmed by PXRD and Raman spectroscopy, which showed no significant structural modification of the samples after reaction with respect to the as-synthesized sample (Figure S4), while the characteristic anhydride bands reached a steady state after the first cycle. To the best of our knowledge, such high photocatalytic stability has not been reported previously for a MOF when this latter is used as a photocatalyst in vapor and in an acidic medium. Interestingly, in sharp contrast with the first cycle, where the H_2_ evolution follows a different profile than the formic acid conversion and CO_2_ production, the second and the third cycles reveal H_2_ production quantities that match the HCOOH conversion and CO_2_ production amounts, reaching the steady state quickly without any significant induction time (Figure 3). This strongly suggests that in the same way as for the formation of anhydride, restructuring of UiO-66-(COOH)_2_-Cu to UiO-66-(COO)_2_-Cu occurs only in the first cycle, and no substantial changes are observed for the following cycles. Moreover, the decrease in H_2_ at the beginning of cycles 2 and 3 is due to a higher surface coverage of the photocatalyst by formic acid in the dark and before a new equilibrium is reached under irradiation conditions at the steady state. The great difference in FAc conversion at the beginning of the dark cycles is due to the adsorption of formic acid rather than its conversion.

**Figure 4:**
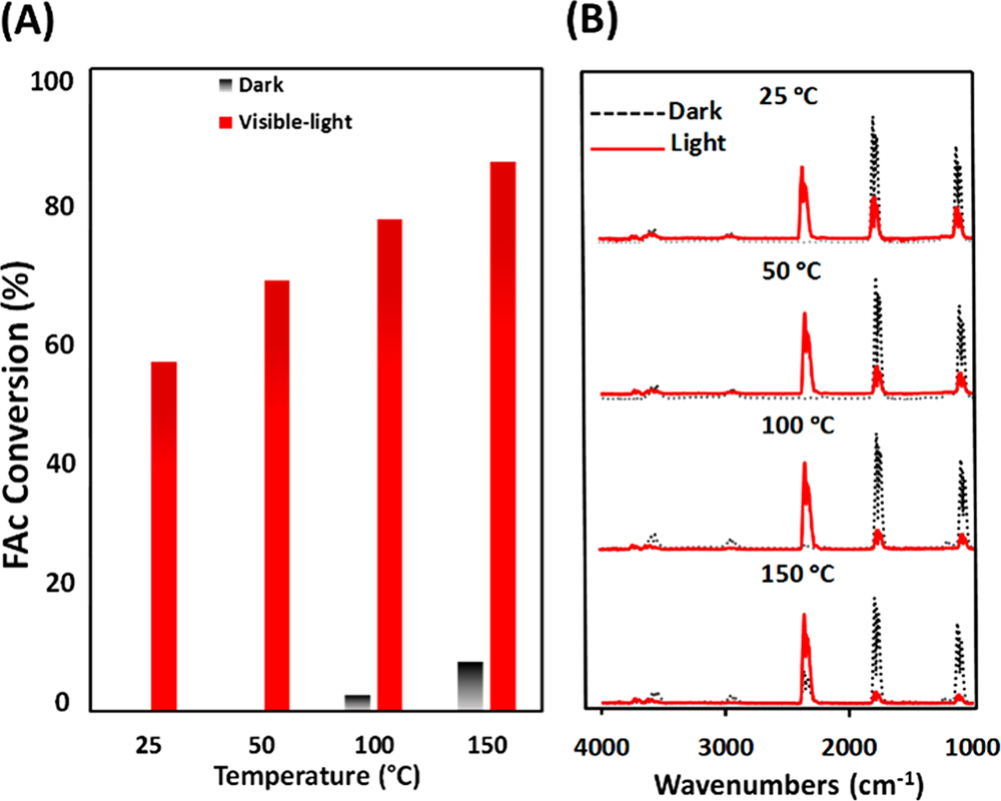
(A) Effect of the temperature on FAc reforming in the dark and under visible-light irradiation. (B) Corresponding IR spectra of the reaction gas phase at the steady state at the studied temperatures between 25 °C (top) and 150 °C (bottom). Reaction conditions: total flow rate = 25 cm^3^·min^–1^; [FAc-^13^C] = 2400 ppm (0.24%) in Ar; 150 W Xe lamp with a visible-light-pass filter (λ > 390 nm); irradiance = 71 mW·cm^–2^; *m*_cat_ = 20 mg (self-supported pellet with a surface area of 1.6 cm^2^).

The thermal activity of UiO-66-(COO)_2_-Cu and the effect of temperature on its photocatalytic performance were further investigated, and the obtained results are shown in Figure 4. As can be clearly seen, no significant thermoactivity is observed in the dark below 100 °C, and the activity is very low between 100 and 150 °C (Figure 4A). However, the temperature increase enhances the photocatalytic activity of the sample under visible light to reach 90% of formic acid conversion at 150 °C while maintaining 100% selectivity with the formation of only CO_2_ and H_2_ (Figure 4B). Nevertheless, after the test performed at 150 °C, the sample was cooled back to room temperature and tested again, and the results were compared with those obtained at room temperature with a fresh sample (Figure S5). A significant drop in the activity was observed, demonstrating low stability of UiO-66-(COO)_2_-Cu at a relatively high temperature in the presence of FAc. The low activity of the sample in the dark demonstrates the main photocatalytic nature of the reaction using UiO-66-(COO)_2_-Cu.

Additionally, the apparent quantum yield for hydrogen production by UiO-66-(COOH)_2_-Cu was determined based on the procedure and equations presented in the Supporting Information and was found to be 10.6% at RT, which is relatively high for a vapor/solid-phase heterogeneous photocatalytic reaction.

### Role of Cu in the Photocatalytic Dehydrogenation

In order to better understand the effect of the copper sites and the UiO-66-(COOH)_2_ framework structure on the photocatalytic activity, Cu-free UiO-66-(COOH)_2_, UiO-66(COOH)_2_-M (M = Ag, Co), and Cu-metalated nonfunctionalized UiO-66 (UiO-66-Cu) were prepared and tested under reaction conditions similar to those for UiO-66-(COOH)_2_-Cu. The Cu-free UiO-66-(COOH)_2_ sample did not show any significant activity under visible-light or UV irradiation (Figure S7). This confirms that the Zr metal centers of UiO-66-(COOH)_2_ have a passive role in the formic acid reforming and can be excluded as catalytically active sites. Moreover, Ag-, and Co-metalated UiO-66-(COOH)_2_ exhibited 4.6- and 6.4-fold lower catalytic activities than the copper-metalated MOF, respectively (Figure S9). With regard to the UiO-66-Cu sample, Cu cations are expected to be inserted in the vicinity of the defect sites of the Zr cluster,^60^ and their content was 8 wt% as determined *via* AAS. The UV spectrum shows similar optical behavior for UiO-66-Cu and UiO-66-(COOH)_2_-Cu (Figure S10A). The photocatalytic test using UiO-66-Cu reveals a much lower activity (8.8% HCOOH conversion) and poorer dehydrogenation selectivity compared to UiO-66-(COO)_2_-Cu (60% *vs* 100%, respectively), as shown in Figure S7. Indeed, these observations indicate that the copper coordinated to the functional groups of UiO-66-(COOH)_2_/UiO-66-(COO)_2_ plays a predominant role in the photocatalytic dehydrogenation of formic acid. Furthermore, the copper content in UiO-66-(COOH)_2_-Cu was varied, and batches containing 6, 9, or 16 wt % copper were tested. It is noteworthy that higher concentration of copper was not possible. Indeed, a maximum of 18% can be achieved for our UiO-66(COOH)_2_ sample, beyond which Cu cations are washed out throughout the purification process. The obtained results are shown in Figures S11 and S12 and demonstrate that the increase in copper content from 6 to 9 wt % is accompanied by an increase in the selectivity as well as an increase in the activity of the MOF from 43% to 47.5%. Finally, a FAc conversion of 65.5% was achieved with the 16 wt % Cu-loaded sample. These results show that even at low copper loading (6 wt %) the activity is still higher than with the other metalated versions (*e.g.*, Co and Ag at higher metal content). More importantly, the 6 wt % UiO-66-(COOH)_2_-Cu is 5-fold more active than the nonfunctionalized system (UiO-66-Cu with 8 wt % Cu), which means that the free carboxylates are thermodynamically favored for copper metalation and formation of active species. Figure S11 also shows that the selectivity is significantly affected at a low Cu loading of 6 wt % where CO was detected with a selectivity of around 10%. Moreover, only a few hundred parts per billion CO is permitted in H_2_ fuel cells in order to prevent poisoning of the catalyst, making the high-Cu-loaded sample the most promising catalyst.^61^

In addition, Cu-MOF-74, which incorporates copper rod secondary building units, and CuO and Cu_2_O nanoparticles were also tested under similar reaction conditions. While no to little activity was observed for CuO and Cu-MOF-74, Cu_2_O showed a low activity (5.6% conversion), indicating the synergic effect between the copper centers and the framework in addition to the importance of the UiO-66-(COOH)_2_ structure in the photocatalytic reaction (Figure S7 and Table S1). Noteworthily, the high catalytic performance, stability, and selectivity of the noble-metal-free UiO-66-(COO)_2_-Cu photocatalyst in the dehydrogenation of formic acid is found to be comparable to those of most homogeneous and heterogeneous photocatalysts reported in the literature (Table S2). However, due to the various possible experimental conditions that can be used for this reaction (irradiation source, light intensity, temperature, irradiated surface of photocatalyst, reactor geometry, mass of the photocatalyst, *etc.*), in addition to missing information in some of the reported works (stability of the catalyst, irradiance, selectivity, *etc.*), we believe that the comparison is mostly qualitative. Nevertheless, an overview of the literature (Table S2) revealed that our work is the first on the reforming of formic acid in the “vapor” phase under “continuous” flow, which is totally innovative and reveals the highest catalytic activity in comparison with other Cu-based photocatalysts under visible light at ambient temperature.

Moreover, the optical band gap energies *E*_g_ for the free and copper-metalated MOFs were determined from the absorbance data using the Tauc plot method (Figure S10B,C), and as expected, the calculated *E*_g_ for UiO-66-(COOH)_2_ was 3.9 eV. However, in the case of UiO-66-(COOH_2_)-Cu (Figure S10C), the *in-situ* formation of Cu(I)–oxo nanoparticles was evident by the narrow band gap observed (*E*_g_ = 1.84 eV), which corresponds to the band gap of Cu_2_O semiconductor.^62^

The charge transfer (CT) between the UiO-66-(COOH)_2_ structure and the copper site was also investigated by transient absorption spectroscopy. The results presented in Figure S13A demonstrate no absorption transient (AT) of the premetalated MOF structure (UiO-66-COOH)_2_, which exhibits strong photoluminescence (PL) (Figure S13C). However, UiO-66-(COOH)_2_-Cu exhibits an AT decay (a broad AT band with a maximum at 440 nm) with very low PL emission (Figure S13B,C), confirming the CT between the MOF structure and the Cu centers. Increasing the FAc concentration led to a leaching of the Cu sites as demonstrated by the decrease in the AT intensity. These results reveal an important role of the MOF structures, beyond being a simple host of the Cu, in the initiation of the *in-situ* structuring of the Cu sites.

Cyclic voltammetry (CV) was performed in 0.5 M Na_2_SO_4_ to reveal the redox reaction of the Cu species in the MOF structure (Figure S14A). CV of UiO-66-(COOH)_2_-Cu depicts redox features at ca. 0.129, 0.015, and −0.643 V *vs* Ag/AgCl, which could be attributed to the redox reactions of copper species inserted into two different coordination sites, the free carboxylate and the defective Zr cluster.^63,64^ UiO-66-(COOH)_2_-Cu shows a higher voltammetric current than UiO-66-(COOH)_2_, indicating a higher conductivity and improved electron/ion mobility. Furthermore, electrochemical impedance spectroscopy (EIS) was employed to gain insight about the CT resistance and the response of the system under various frequency regimes.^65^ UiO-66-(COOH)_2_-Cu shows smaller CT resistance than UiO-66-(COOH)_2_ (Figure S14B), which confirms the enhanced conductivity and better electron mobility in UiO-66-(COOH)_2_-Cu.

### Restructuring Phenomena as Investigated by *Operando* Analysis of the Photocatalyst Surface

**Figure 5:**
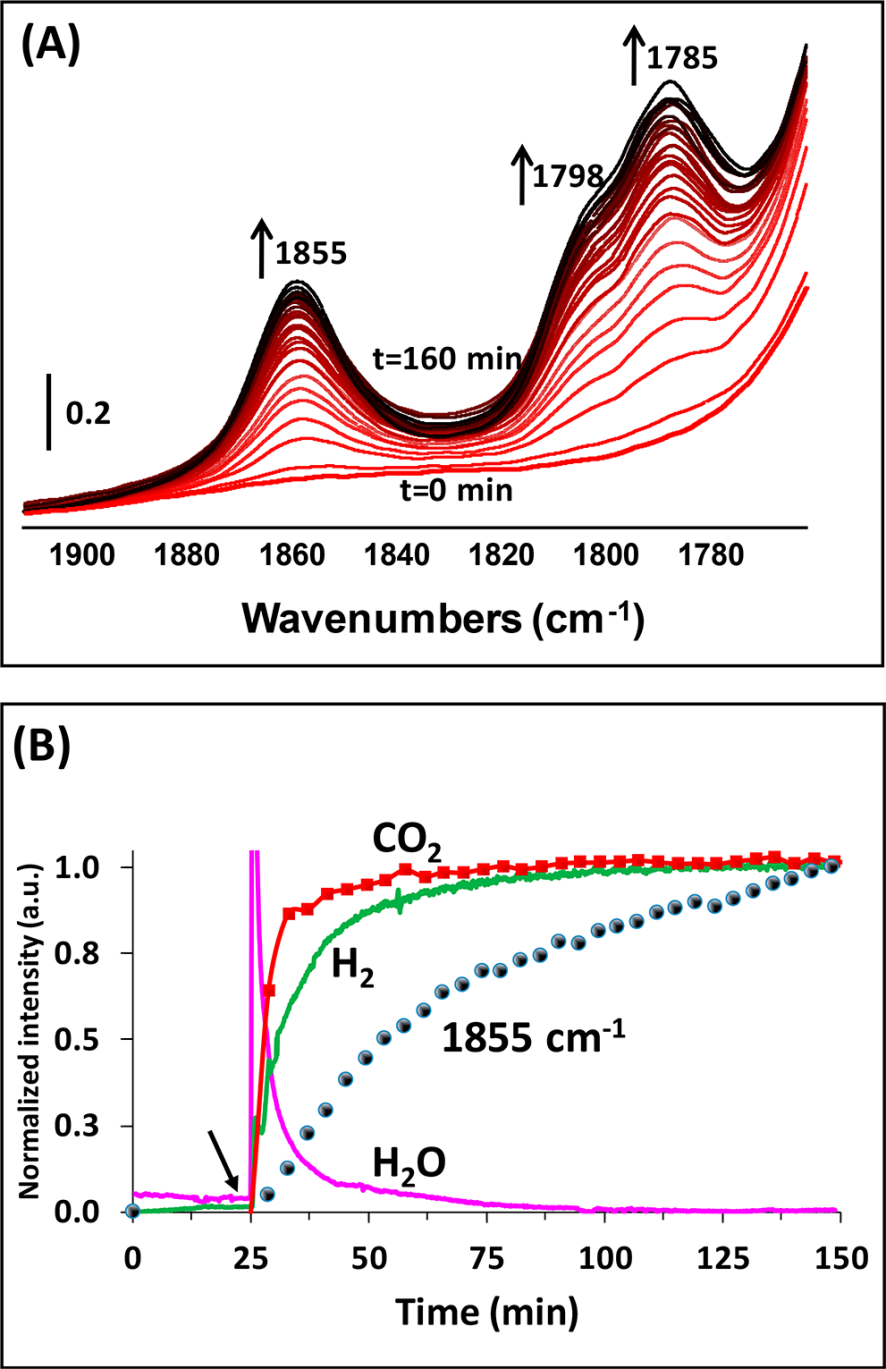
(A) FTIR spectra of the UiO-66-(COO)_2_-Cu surface during the reforming of formic acid-^13^C under visible-light irradiation. (B) Evolution of the corresponding normalized intensities of the gas-phase products and the band area at 1855 cm^–1^ of the surface *versus* the irradiation time. Reaction conditions: total flow rate = 25 cm^3^·min^–1^; [formic acid-^13^C] = 2400 ppm (0.24%) in Ar; *T* = 25 °C; 150 W Xe lamp with a visible-light-pass filter (λ > 390 nm); irradiance = 71 mW·cm^–2^; *m*_cat_ = 20 mg (self-supported pellet with surface are of 1.6 cm^2^). The arrow in (B) corresponds to the light-on time.

The evolution of the IR spectra of the photocatalyst surface in real time was monitored simultaneously with the gas-phase analysis, as previously mentioned using the IR *operando* setup.^66^ As can be seen in Figure 5A, a gradual increase in the intensities of the bands at 1855, 1798, and 1785 cm^–1^ with the reaction time was observed for UiO-66-(COOH)_2_-Cu, which corresponds to the anhydride formation (UiO-66-(COO)_2_-Cu). In addition, water released from the catalyst surface was simultaneously detected. In general, water molecules could be produced from the dehydration process of the formic acid. Nevertheless, the absence of CO in the gas phase suggests another origin of the water release, which most likely results from the dehydration process of the carboxylate functions prior to the formation of bridging anhydrides in the first few minutes of irradiation as well as of the copper clustering, which will be discussed further in the XPS and HRTEM analysis section. Once the anhydrides reach a steady state, the hydrogen production also attains its steady state (Figure 5B). However, this trend was only observed in the first cycle, showing the irreversible behavior of this restructuring process that leads to the formation of the highly active UiO-66-(COO)_2_-Cu photocatalyst.

**Figure 6:**
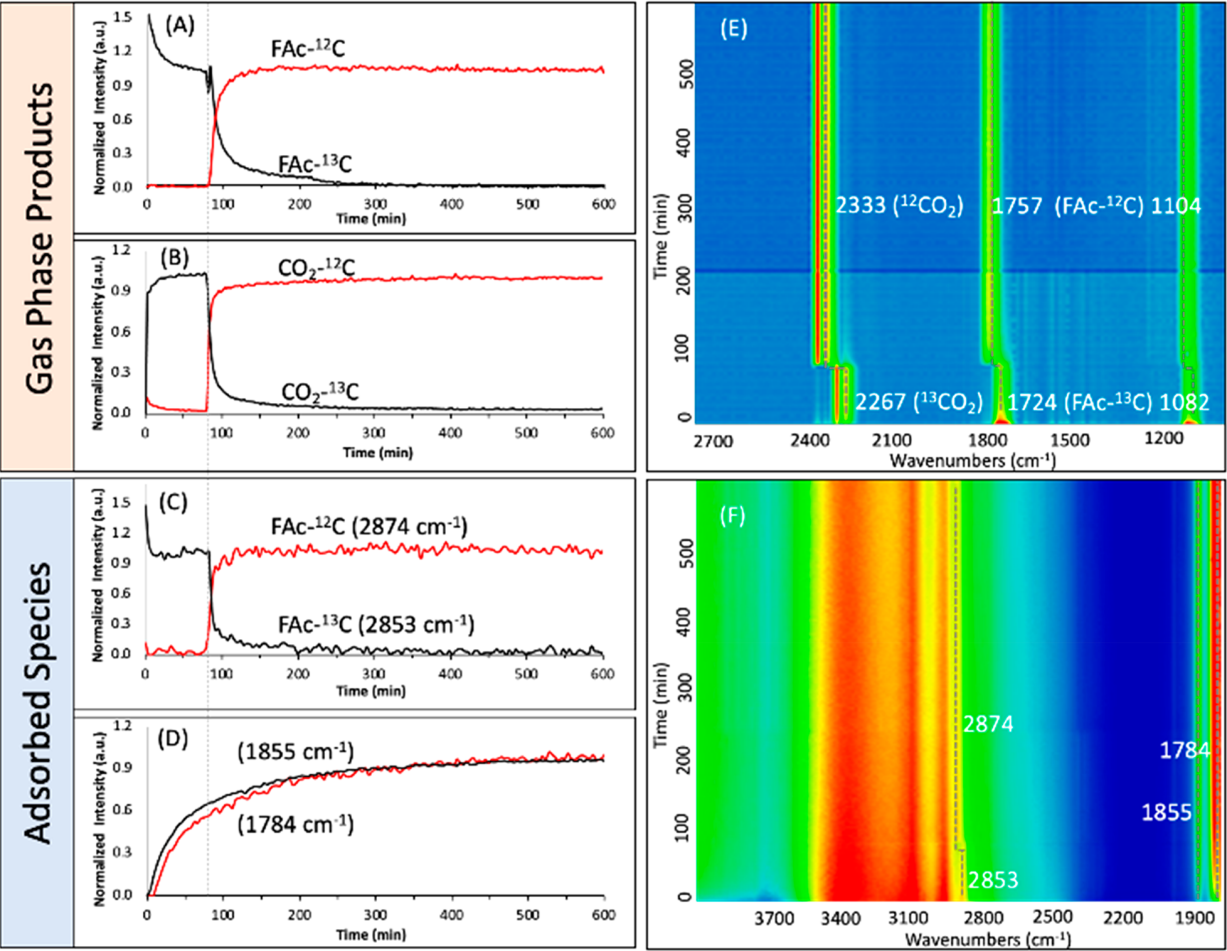
(A–D) Evolution of (A, B) gas-phase products and (C, D) adsorbed species on UiO-66-(COO)_2_-Cu *versus* time in the FAc-^13^C/FAc-^12^C SSITKA experiment (*t* = 0 corresponds to the start of irradiation, and the dotted line corresponds to the FAc-^13^C/FAc-^12^C). (E, F) Relative evolution of the IR intensities from lower (blue color) to higher (red color) for (E) the reaction gas phase and (F) the photocatalyst surface. Reaction conditions: total flow rate = 25 cm^3^·min^–1^; [FAc-^13^C] = [FAc-^12^C] = 2400 ppm (0.24%) in Ar; *T* = 25 °C; 150 W Xe lamp with a visible-light-pass filter (λ > 390 nm); irradiance = 71 mW·cm^–2^; *m*_cat_ = 20 mg (self-supported pellet with a surface are of 1.6 cm^2^).

To gain a deep understanding of the process for restructuring of UiO-66-(COOH)_2_-Cu to UiO-66-(COO)_2_-Cu and the formation of the anhydride, a steady-state isotopic transient kinetic analysis (SSITKA) experiment using *operando* FTIR spectroscopy was performed. It corresponds to replacing the formic acid by its isotope in the steady state under the same reaction conditions. This isotopic transient would induce a shift/modification of the mass spectrometry signals and/or the IR vibration bands of the corresponding products as well as of the species adsorbed on the analyzed surface. Therefore, as the IR bands of the carboxylate functions of the formic acid and the UiO-66-(COOH)_2_-Cu ligands could overlap, this experiment allows the origins of the final products and the anhydride to be distinguished. The so-obtained results are shown in Figure 6 and illustrate the kinetics of the reaction in the gas phase and the adsorbed surface species during the SSITKA experiment. In contrast to the shift and isotopic exchange observed for the gas-phase products (Figure 6 A,B,E) and for the adsorbed formic acid (Figure 6C,F), no perturbation of the band positions at 1855 and 1784 cm^–1^ is observed (Figures 6D,F and S15). These results indicate that the anhydride formation resulted from selective cross-coupling of the MOF carboxylate linkers. Nevertheless, these bands are not observed during the preactivation of the photocatalyst under an inert carrier gas and are detected only during the reaction. Therefore, an indirect role of the formic acid in the anhydride formation cannot be excluded. In addition, the corresponding IR bands of anhydride functions were not observed when Cu-free UiO-66-(COOH)_2_ was employed as the photocatalyst under similar reaction conditions, which clearly emphasizes the role of Cu in assisting the formation of anhydride in UiO-66-(COO)_2_ under visible light. Our hypothesis is that HCOO^–^H^+^ delivers protons to the metalated carboxylate (COO–Cu−) functions of the ligand, promoting the formation of those anhydrides under irradiation. Furthermore, the positions of these characteristic bands are shifted toward lower ν values compared to the free anhydrides formed by thermal treatment of UiO-66-(COOH)_2_ due to the insertion of copper cations.^54^

**Figure 7:**
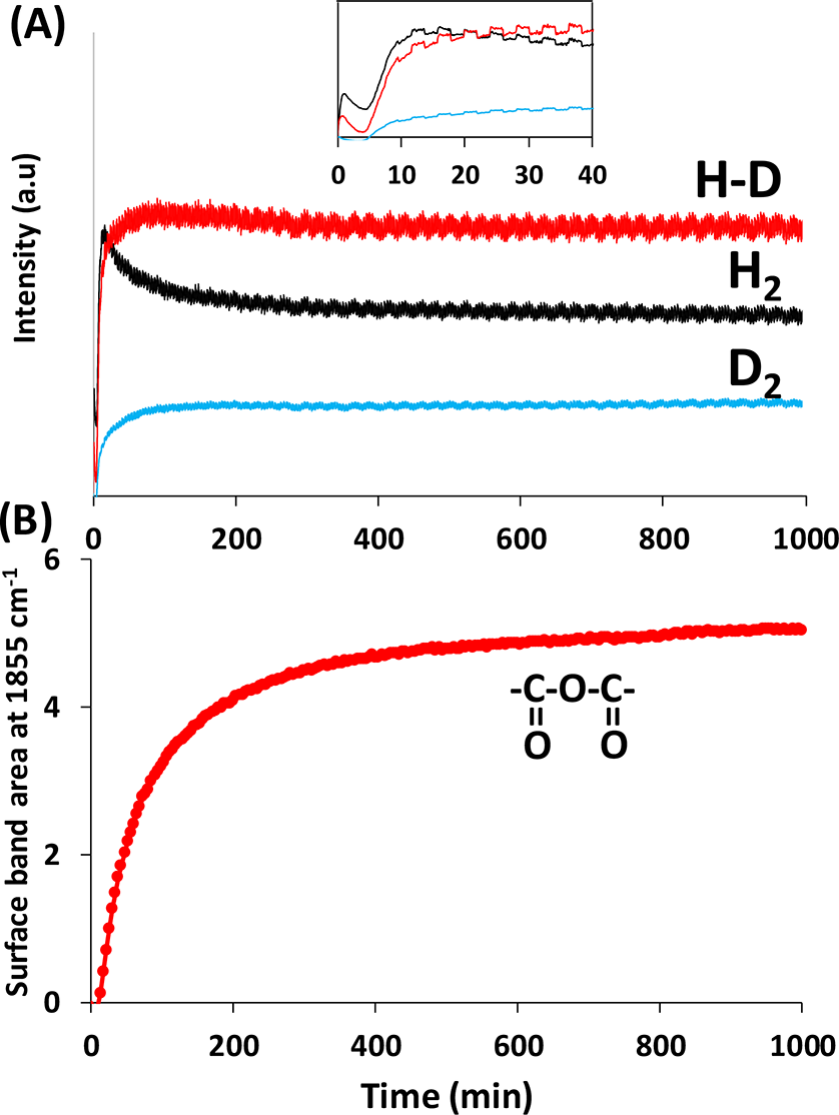
Evolution of (A) the hydrogen isotopes and (B) the anhydride vibration bands during the photocatalytic dehydrogenation of DCOOH under visible light. The inset in (A) is a zoomed-in view of the first 40 min of the reaction. Reaction conditions: total flow rate = 25 cm^3^·min^–1^; [DCOOH] = 2400 ppm (0.24%) in Ar; *T* = 25 °C; 150 W Xe lamp with a visible-light-pass filter (λ > 390 nm); irradiance = 71 mW·cm^–2^; *m*_cat_ = 20 mg (self-supported pellet with a surface area of 1.6 cm^2^).

The dehydrogenation of DCOOH was further explored in order to gain insight into the hydrogen evolution during the first minutes of irradiation. The formation of DH is expected as the main product of the dehydrogenation of DCOOH in the case where the dissociation takes place on a single site. However, the obtained results depicted in Figure 7A show a mixture of hydrogen isotopes with a ratio of H_2_/HD/D_2_ swapped from 0.46/0.43/0.11 after 20 min of reaction to 0.35/0.5/0.15 in the steady state. The generation of H_2_ and D_2_ confirms that the dissociation occurs in multiple steps and/or on multiple sites. This can be attributed to the scrambling of the formate and formyl groups, as we demonstrated in our previous work in the case of methanol photooxidation.^67^ In addition, the evolution of the statistical values of the isotope ratios follows a similar trend as the anhydride bands (Figure 7B), suggesting a different mechanism for DCOOH decomposition in the steady state with respect to that at the beginning of the reaction, which is coherent with the restructuring of the catalyst.

### Restructuring Phenomena as Investigated by XPS and HRTEM-EDX Analyses

More insight into the restructuring process of the catalyst during the reaction was obtained from SEM-EDX mapping, AAS, XPS, and HRTEM-EDX mapping analyses of the material before and after the photocatalytic reaction tests. The SEM images and EDX mapping of the samples before and after reaction (Figure S16) demonstrate a small modification of the gradual concentration of copper in the framework. However, no modification of the Cu/Zr ratio (0.75) and copper content (19 wt %) was detected, in line with the elemental analysis of the samples by AAS (Figure S17). On the other hand, the XPS analysis of the two samples demonstrates a very significant (around 8-fold) decrease in the Cu/Zr ratio from 4.1 to 0.5 after the reaction (Figures S18 and S19). This deviation between the EDX and XPS analysis data could be explained by the limited penetration of the XPS beam in the samples (only a few nanometers) while the EDX and AAS analyses are more global.

**Figure 8:**
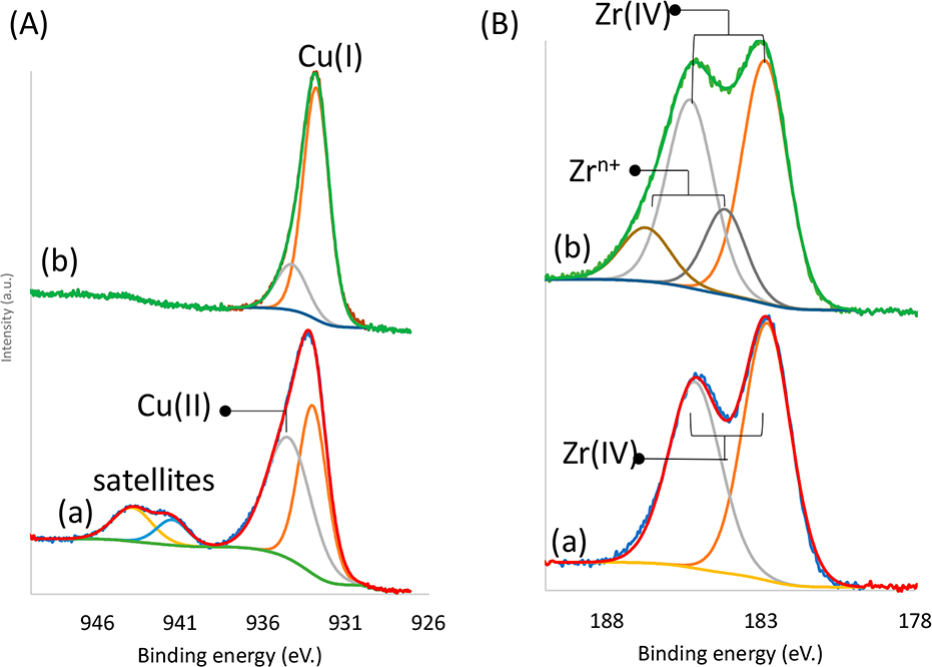
(A) Cu 2p_3/2_ and (B) Zr 3d levels of UiO-66-(COO)_2_-Cu (a) before and (b) after reaction.

Furthermore, XPS analysis was performed for UiO-66-(COOH)_2_ and its metalated form before and after reaction (Figures 8, S18, and S19). The obtained data reveal information on the evolution of the oxidation state of the copper. The Cu 2p_3/2_ peak at 935.8 eV observed for UiO-66-(COO)_2_-Cu could be assigned to Cu(II), with shake-up peaks of 2p to 3d observed between 940–945 eV, and the more negative peak at 933 eV could be ascribed to Cu(I) and was the main detected peak in the sample after reaction. The latter was assigned to Cu_2_O formation, with a quasi-total disappearance of the highly coordinated Cu(II) species. Therefore, the absence of the Cu 2p satellite clearly indicates that after the reaction there are mainly Cu(I) species present (Figure 8). The results also demonstrate some perturbation of the range of Zr as well, which could be mainly assigned to some modifications of the environment of the Zr sites. However, as the analysis was performed *ex-situ*, it is not possible to discuss the results quantitatively because the state of the catalyst surface could have changed after exposure to the atmosphere (*e.g.*, oxidation of the metal clusters). We strongly believe that the photogenerated electrons promote the reduction of Cu(II) to form lower-oxidation-state species (*e.g.*, Cu(I) and/or Cu(0)) during the reaction. The presence of the latter was confirmed by the diffuse reflectance spectra recorded for the UiO-66-(COO)_2_-Cu pellets freshly taken after the reaction, which showed the typical broad absorption band of Cu_2_O in the visible region (420 and 485 nm) in addition to a shoulder peak evolved at 625 nm mainly in the highly loaded sample, which corresponds to the plasmonic band of Cu NPs^68^ (Figure S20).

**Figure 9:**
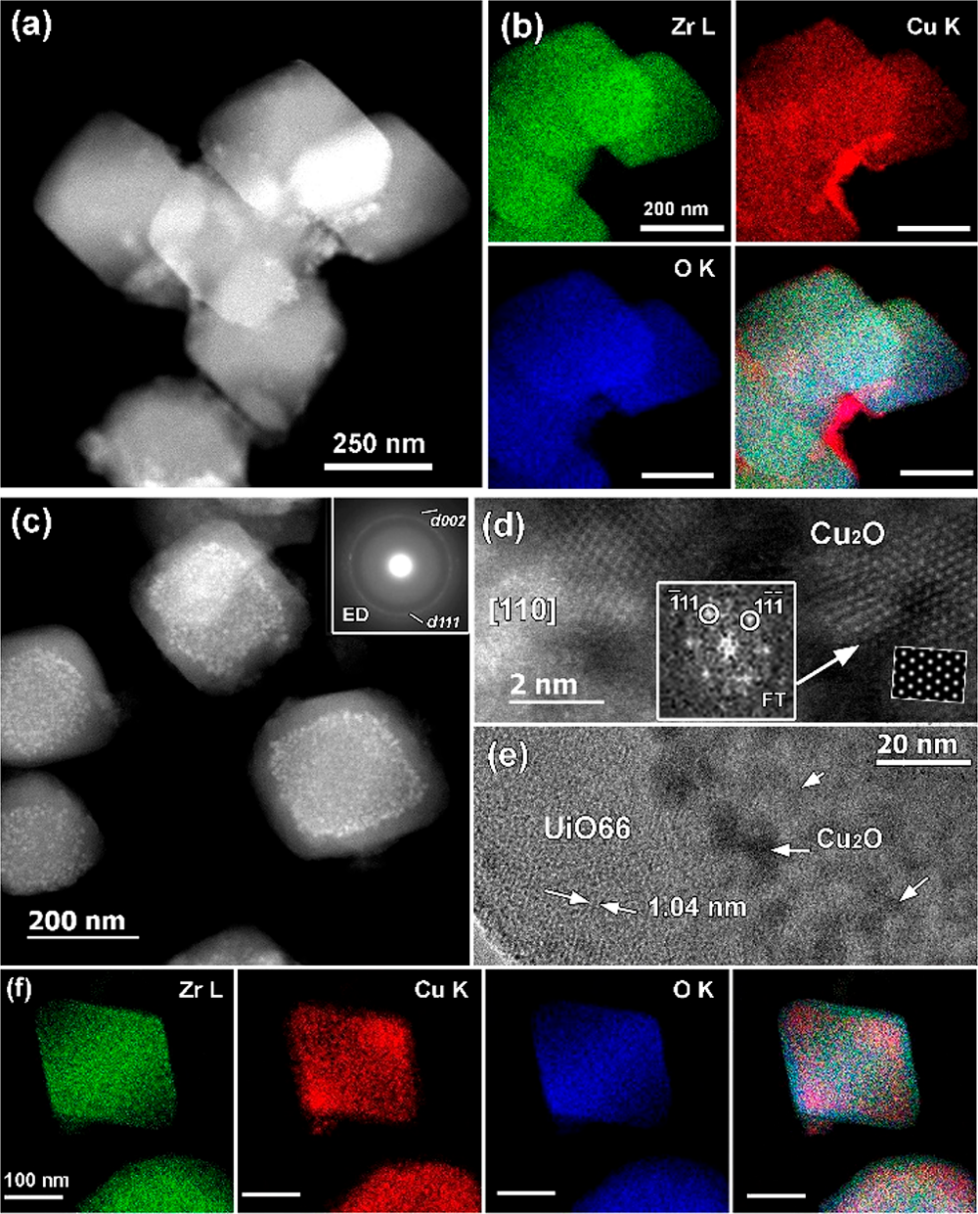
(a) HAADF-STEM image of UiO-66-(COOH)_2_-Cu material and (b) corresponding EDX-STEM elemental mappings for Zr L (green), Cu K (red), and O K (blue) and the overlaid color image before reaction. (c) HAADF-STEM image of UiO-66-(COO)_2_-Cu obtained after reaction with the corresponding ring SAED pattern indexed based on the cubic *Pn3̅m* structure of Cu_2_O. (d) High-resolution HAADF-STEM image of Cu-based nanoparticles formed after the reaction and assigned to Cu_2_O. The inset shows the corresponding [110] FT pattern indexed based on the cubic Cu_2_O cubic structure, and the simulated [110] HAADF-STEM image in the white box shows a good fit to the experimental image. (e) Bright-field HRTEM image of the edge of a UiO-66-(COO)_2_-Cu nanoparticle after reaction. The Cu_2_O NPs exhibit black contrast, marked with white arrows. The Cu-free near surface region of UiO-66-(COO)_2_-Cu nanoparticles should be noticed. (f) EDX-STEM elemental mappings of UiO-66-(COO)_2_-Cu particles after reaction for Zr L (green), Cu K (red), and O K (blue) and the overlaid color image showing the diffusion of the copper element inside the UiO-66-(COO)_2_-Cu framework.

Transmission electron microscopy shed more light on the zoning phenomenon of the copper before and after reaction. The obtained high-angle annular dark-field scanning TEM (HAADF-STEM) images in combination with STEM-EDX elemental mapping depicted in Figure 9a,b confirm a higher concentration of the copper clusters/atoms at the external surface of the UiO-66-(COOH)_2_-Cu crystal before the reaction, demonstrating limited diffusion of the Cu(II) into the MOF. After reaction, the formation of copper clusters inside the crystal with sizes of around 3–5 nm (core–shell form) can be clearly observed in the HAADF-STEM image (bright contrast) (Figure 9c) and STEM-EDX elemental mapping (Figure 9f). Although metal sintering from the nanopores of a crystalline porous structure to its external surface is a very usual phenomenon that has already been observed in the case of zeolites, to our knowledge the opposite migration has not been reported previously. Moreover, no diffraction index of the CuO clusters was detected, in agreement with the PXRD data. The selected-area electron diffraction (SAED) pattern taken from single UiO-66-(COO)_2_-Cu clusters after reaction revealed weak diffraction rings (Figure 9c inset) which can be indexed based on the cubic Cu_2_O structure (*Pn*3̅*m*, *a* = 0.426 nm; ICSD 172174). This could be attributed to the Cu–oxo-like species formed during the *in-situ* restructuring of Cu(II) during the reaction and stabilized by an electrostatic interaction with the remaining carboxylate defects of the structure. Nevertheless, no *ex-situ* detection of Cu(0) NPs was observed, probably because of their high dispersion and low stability after exposure of the sample to air. However, a similar *in-situ* restructuring process was recently demonstrated for the CuO–TiO_2_ and CuO–Nb_3_O_7_(OH) systems.^69^ In these hetero-nanostructures, the nonactive CuO–TiO_2_ and CuO–Nb_3_O_7_(OH) undergo reduction reactions under light irradiation to form the active Cu_2_O–TiO_2_ and Cu(0)–Nb_3_O_7_(OH), respectively. The formation of Cu(0) is demonstrated as well by the use of Cu_2_O, which exhibits an induction time of 2 h assigned to the *in-situ* formation of Cu(0) NPs. The copper clustering process is in agreement with the release of water detected by *operando* analysis, which could be assigned to dehydration of both carboxylate and CuOH groups. Furthermore, the high-resolution HAADF-STEM imaging (Figure 9d) and high-resolution TEM (HRTEM) (Figure 9e) confirmed the Cu_2_O structure of the clusters and UiO-66 structure of the framework. The image of the 002 lattice planes in the shell of the UiO-66-(COO)_2_-Cu particle (Figure 9e) suggests that the UiO-66 structure stays intact after Cu diffusion. Moreover, the analysis of the UiO-66-Cu sample used as a reference, in which copper is anchored within the Zr-defective clusters, demonstrates total deterioration of the crystal after only a few hours of reaction (10 h) (Figure S21). Total structure destruction accompanied by the formation of Cu nanoparticles was confirmed by the PXRD pattern of the UiO-66-Cu sample after reaction (Figure S22). These interesting results reveal the crucial role of the bridged anhydrides in preserving the UiO-66-(COO)_2_-Cu crystallinity and therefore justify the high stability of this photocatalyst. In addition, the anhydride bridges could form cagelike structures around copper clusters, preventing the migration of the particles and further stabilizing the UiO-66 structure.

### Complementary Microscopic Insight into the Reaction Mechanism by DFT Calculations

**Figure 10:**
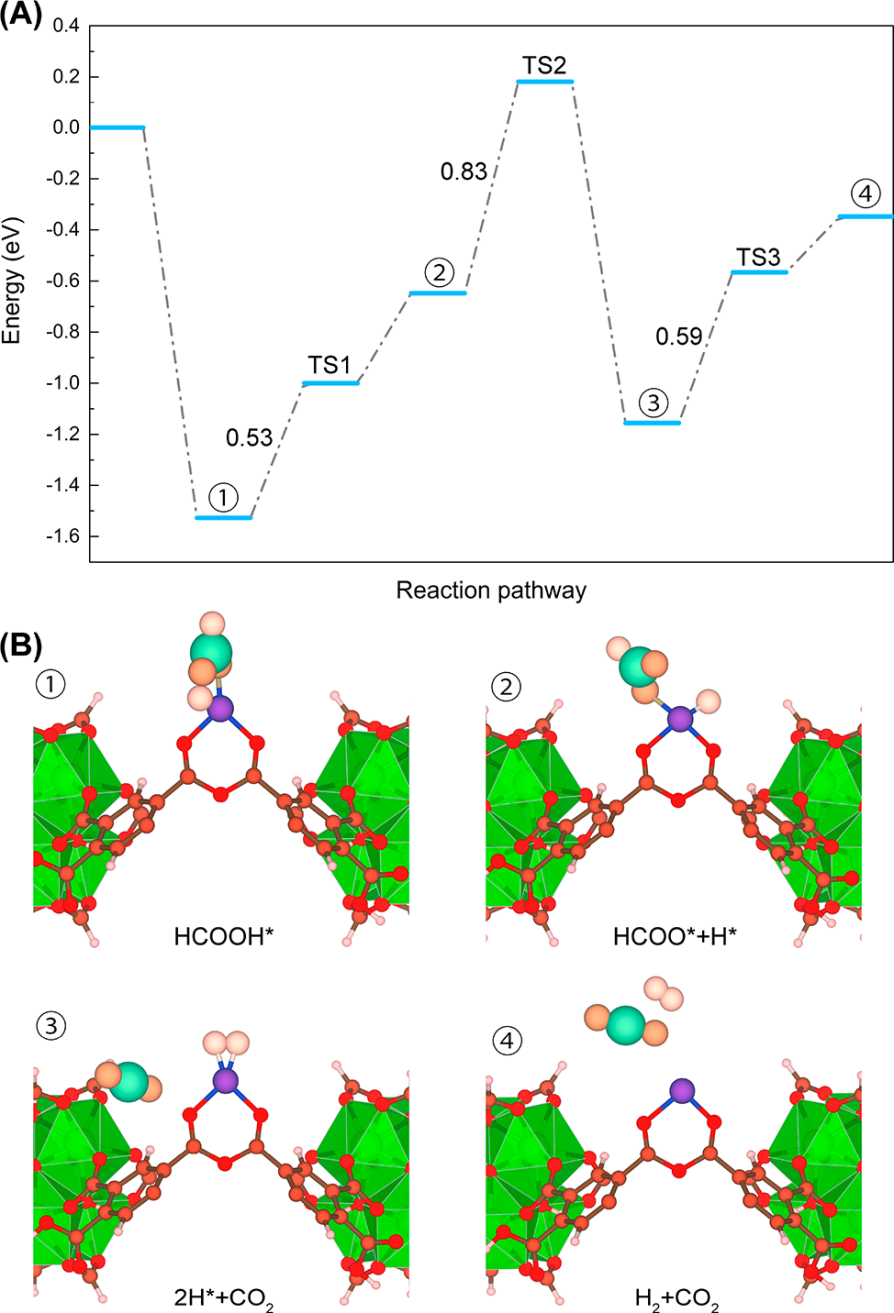
(A) DFT-derived minimum-energy pathway for the dehydrogenation of formic acid by UiO-66-(COO)_2_-Cu and (B) corresponding illustrative snapshots of the different intermediate species. The energy barriers (Enthalpy, expressed in eV) for the three transition states (TSs) are also shown in the figure. Color codes for the MOF: C, gray; Cu, blue; O, red; H, white; Zr, green. Color codes for the adsorbed molecules: C, light blue; O, orange; H, white. The total free energy of the UiO-66-COO)_2_-Cu structure with a gas-phase HCOOH molecule was set to zero in the Gibbs free energy profile.

DFT calculations first explored the preferential geometry of the UiO-66-(COO)_2_-Cu system starting with the experimental conclusions that Cu assists the dehydration of the carboxylic groups. Figure S1 reports a representation of the two-coordinate Cu(I) configuration with the surrounding oxygen atoms of the anhydride group in the DFT-optimized cluster model of UiO-66-(COO)_2_-Cu. Due to the complexity of the structure, only a single copper site and its environment were considered in these calculations. It is noteworthy that this modeled complex was observed experimentally through the *in-situ* FTIR measurements discussed earlier. As a further step, the minimum-energy reaction pathway was explored for the dehydrogenation of formic acid by UiO-66-(COO)_2_-Cu throughout the HCOO dehydrogenation (Figure 10A) and COOH dehydrogenation (Figure S23) routes. The first step of the HCOO dehydrogenation reaction proceeds *via* the coordination of the HCOOH molecule toward the Cu site (Figure 10B), which is associated with a high adsorption energy of −1.53 eV and a large fraction of electrons accumulated along the Cu–O(CO) bond (Figure S24). The adsorbed HCOOH molecule (labeled as HCOOH*) then undergoes O–H bond cleavage *via* the formation of a first transition state (TS1) corresponding to an energy barrier of 0.53 eV that leads to the formation of HCOO* and H*. This intermediate species evolves toward a second transition state (TS2) with an energy barrier of 0.83 eV prior to forming CO_2_ as well as 2H*, which subsequently dehydrogenates *via* a third transition state (TS3) accompanied by an energy barrier of 0.59 eV to further release a gas-phase H_2_ molecule.

**Scheme 1:**
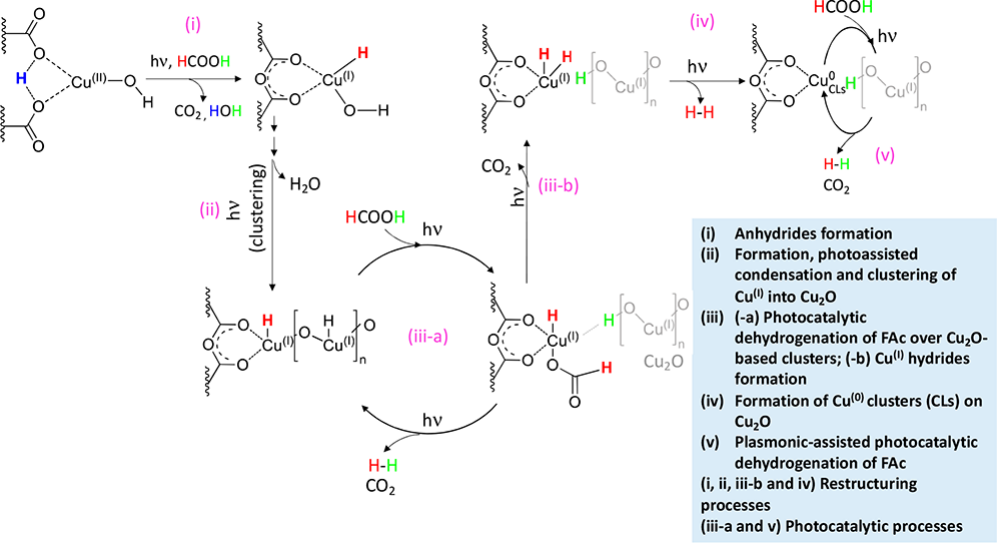
Plausible Restructuring Pathways of UiO-66-(COOH)_2_-Cu during FAc Reforming under Visible Light Leading to the Formation of the Supported and Stabilized Cu(0)/Cu(I) Binary System on UiO-66-(COO)_2_

In the COOH dehydrogenation pathway, the adsorption configuration of the HCOOH molecule corresponds to a metastable state with the corresponding adsorption energy of −1.27 eV compared to the adsorption configuration of the HCOOH molecule in the HCOO dehydrogenation mechanism (Figures S23 and S24). Next, the intermediate species COOH* and H* are formed *via* C–H bond breaking (TS1 with an energy barrier of 1.13 eV) followed by the formation of COOH** *via* TS2 (with an energy barrier of 0.58 eV) and its further transfer to HCOO′ and H*. Then HCOO′ dehydrogenates *via* TS3 with a barrier of 1.12 eV to produce intermediate CO_2_ + 2H*. Finally, the adsorbed H* intermediate is released from the Cu site to form H_2_. For the HCOO dehydrogenation pathway, the rate-determining step (RDS) is the transformation of HCOOH* + H* to 2H* + CO_2_ with an energy barrier of 0.83 eV, while the formation of H* + HOOC is the RDS for the COOH dehydrogenation, with an energy barrier of 1.13 eV. This observation suggests that the HCOO dehydrogenation pathway is more plausible. Remarkably, in the final product, Cu still keeps its original two-coordinate geometry and its +1 oxidation state to further adsorb another HCOOH molecule to initiate a second dehydrogenation cycle, which is also consistent with what was observed experimentally. It should be noted that the Cu clusters could coordinate with the formate of the formic acid as well as with the unbridged carboxylates of the MOF structure. An alternative plausible pathway can be suggested based on our experimental findings, and it is strongly related to the reaction mechanisms reported on traditional hetero-nanostructured photocatalysts (Scheme 1). After the formation of anhydride–Cu(I) during the reaction, a nanoclustering process leads to the formation of Cu_2_O NPs, in agreement with the observation of these NPs during the HRTEM and XPS analysis of the samples after reaction and the release of water detected by *operando* analysis. The surface Cu(I) particles undergo further reduction through the photogenerated electrons, which results in the formation of Cu(0) clusters (Cu^0^_CLs_) that act as an electron pool for the reduction of protons into H_2_. Although Cu^0^_CLs_ are not effective in photocatalysis when they are isolated as single metal particles, their photoactivity in our system can be explained by the photosensitization and/or charge transfer pathways through their strong interactions with the anhydride units and with Cu_2_O nanostructured surface. In addition, the anhydride functions play a crucial role as stabilizers of the Cu and Cu(I)–oxo particles during the reaction according to the notable high stability of the UiO-66-(COO)_2_-Cu photocatalyst.

## Conclusion

A copper-metalated metal–organic framework, namely, UiO-66-(COO)_2_-Cu, was engineered, fully characterized, and employed as a photocatalyst for the dehydrogenation of formic acid. This noble-metal-free catalyst was demonstrated to be highly selective (>99.99%), stable (3 days of reaction), and efficient at room temperature with a high formic acid dehydrogenation yield (5 mmol·g_cat_^–1^·h^–1^). The high performance and stability of UiO-66-(COO)_2_-Cu compared with the standard copper-metalated UiO-66-Cu was attributed to the *in-situ* restructuring process that takes place at the surface of the former through intraframework cross-linking, resulting in the formation of highly active Cu^0^/Cu_2_O NPs trapped in the UiO-66 cages. This study opens the way for the design of new Cu-based MOFs for various applications in photocatalysis. It allows highlighting of the mechanism of formic acid dehydrogenation, a potential intermediate in several reactions such as CO_2_ reduction.

## Supporting Information Available

Synthesis and characterization of the MOF catalysts, including PXRD patterns, UV–vis and FTIR spectra, Raman spectra, SEM-EDX mapping, atomic absorption, XPS, CV, and EIS analyses, and DFT calculations (PDF)
