# Supplementary material for: Selective Photocatalytic Dehydrogenation of Formic Acid by an In Situ-Restructured Copper-Postmetalated Metal–Organic Framework under Visible Light
Source: J Am Chem Soc. 2022 Sep 1;144(36):16433–46. doi: 10.1021/jacs.2c04905 (PMC9479070; doi:10.1021/jacs.2c04905)
Supplement: Supplementary file 1 — ja2c04905_si_001.pdf [file ja2c04905_si_001.pdf]

Selective Photocatalytic Dehydrogenation of Formic Acid by an *In Situ*-Restructured Copper-Postmetalated Metal–Organic Framework under Visible Light

Houeida Issa Hamoud,[†] Patrick Damacet,[‡] Dong Fan,[‡‡] Nisrine Assaad,[‡] Oleg I. Lebedev,[††] Anna Krystianiak,[‡†] Abdelaziz Gouda,[⊥] Olivier Heintz,[‡†] Marco Daturi,[†] Guillaume Maurin,[‡‡] Mohamad Hmadeh,[*,‡] and Mohamad El-Roz,[*,†]

[†] Normandie Univ, ENSICAEN, UNICAEN, CNRS, Laboratoire Catalyse et Spectrochimie, 14050 Caen, France

[‡] Department of Chemistry, American University of Beirut, P.O. Box 11-0236, Riad El-Solh, Beirut 1107 2020, Lebanon

[††] Normandie Univ, ENSICAEN, UNICAEN, CNRS, Laboratoire CRISMAT, UMR 6508, 14050 Caen, France

[⊥] Department of Chemistry, University of Toronto, 80 St. George Street, Toronto, ON M5S 3H6, Canada

[‡‡] Institut Charles Gerhardt Montpellier (ICGM), University of Montpellier, CNRS, ENSCM, 34095 Montpellier, France

[‡†] ICB, CNRS UMR 6303 – Université de Bourgogne Franche-Comté, 9 Avenue A. Savary, 21078 Dijon, France

Synthesis of UiO-66-$(COOH)_2$

UiO-66$(COOH)_2$ was synthesized under conditions similar to those reported in the literature[1]. In brief, an equimolar amount of zirconyl chloride octahydrate (59.4 mg, 0.184 mmol) and 1,2,4,5-benzenetetracarboxylic acid (47.1 mg, 0.184 mmol) were dissolved in 4 mL of DMF already placed in a 20 mL scintillation vial and sonicated for a couple of minutes. 4 mL of formic acid modulator was later added to the obtained mixture followed by sonication for few extra minutes. The reaction mixture was then placed in a pre-heated oven at 130^0C for 5 hours. The obtained white powder was collected by centrifugation at 4000 rpm and washed five times with DMF and three times with MeOH. UiO-66$(COOH)_2$ was then dried under dynamic vacuum oven at 110^0C overnight**.**

Synthesis of UiO-66

UiO-66 was prepared by a similar synthesis route previously reported in literature[2]. In brief, zirconium (IV) chloride (1.48 mmol, 344 mg) and terephthalic acid (1.48 mmol, 245 mg) were dissolved in a solution containing 40 mL of DMF and 0.1 mL of DI and sonicated for 10 minutes. 11.13 mL of formic acid modulator (200 equivalents) was added to the mixture and further sonicated for 15 minutes. The reaction mixture was then placed in a preheated oven for 21 hours at a temperature of 120°C to produce the UiO-66 crystals. Finally, the crystals were washed with DMF several times over a period of three days, activated by solvent exchange with DCM, and lastly dried under vacuum for 12 hours at 125°C.

Synthesis of UiO-66-$(COO)_2$-Cu

In a 20 mL scintillation vial, 30 mg of copper nitrate was dissolved in 15 mL of DMF by sonication for 10 mins until a clear solution was obtained. 60 mg of UiO-66$(COOH)_2$ was then added to the copper solution and sonicated for few minutes to ensure full MOF dispersion in the solution. The

mixture was then stirred on a hot plate at 75°C for 21 hours until brownish microcrystalline powder was obtained. The supernatant was discarded by centrifugation and the solids were washed with DMF for 2 days with fresh DMF being exchanged 3 times per day followed by fresh methanol for another 2 days. Finally, the solids were collected by centrifugation and dried in a vacuum oven at 80°C overnight.

Synthesis of UiO-66-$(COO)_2$-M

UiO-66-(COO_2)-M (M = Ag, Co) was prepared following the same synthesis procedure used for UiO-66-$(COO)_2$-Cu using silver nitrate, and cobalt nitrate hexahydrate instead of copper nitrate as a metal source in the post metalation process

Synthesis of UiO-66-Cu

UiO-66-Cu was prepared following the same synthesis procedure used for UiO-66-$(COO)_2$-Cu using the unfunctionalized UiO-66 instead of UiO-66-$(COOH)_2$.

Synthesis of copper-based MOF-74

Cu-MOF-74 was prepared according to a literature procedure previously reported[3]. Briefly, a mixture of trihydrated copper nitrate and 2,5-dihydroxy terephthalic acid were dissolved in DMF, sonicated for few minutes, and later transferred to a 100 mL reactive vial. Few mL of 2-propanol was then added to the mixture that was in turn placed in a preheated oven at 100°C for 18 hours. The resulting reddish microcrystalline powder was washed with DMF for two days, followed by methanol for another two days and finally placed in a vacuum oven at 70°C overnight.

Determination of copper content in UiO-66-Cu and UiO-66-$(COO)_2$-Cu via AAS

The MOF samples were digested by dissolving 0.5 mg of each copper-loaded MOF in 200μL aqua regia mixture of Nitric acid (65%) and hydrochloric acid (36%), in addition to 200μL of hydrofluoric acid. Following sonication, 10mL of deionized water was added to dilute the solution

resulting in a total volume of 10.4 mL. Copper weight percentage was determined via an iCE 3000 series atomic absorption spectrophotometer using an air-acetylene flame as a fuel source and was calculated using the below formula:

$$Cu\ Weight\ \% = \frac{C_{Cu} x V_{Solution}}{m_{MOF}} x100$$

Where C_{Cu} is the copper's concentration in the MOF sample in ppm determined via AAS, $V_{Solution}$ is the total volume of the mixture in L, and m_{MOF} is the mass of the Cu-MOF sample used in mg.

Calculation of apparent quantum yield of UiO-66-(COO)$_2$-Cu

The apparent quantum yield for H_2 production was determined using the following equation:

$$\mathbf{\Phi H2} = \frac{\mathbf{2\times molecules\ of\ hydrogen\ evolved/cm2}}{\mathbf{number\ of\ incident\ photons/cm2}}\ \mathrm{x}\ f$$

With $f = \frac{\mathrm{S(irradiated\ pellet)}}{\mathrm{S(reactor\ used\ for\ calculation\ of\ }\boldsymbol{\Phi})} = \frac{1.6\ cm2}{12.5\ cm2} = 0.128$

The produced H_2 amount was quantified by FTIR and the incident photons were measured using $K_3Fe(C_2O_4)_3$ as chemical actinometer. Thus, 3mL (V_1) of an aqueous solution containing the iron actinometer (0.15 M) and H_2SO_4 (0.05 M) was prepared in the same reactor used in the photocatalytic tests. After irradiation with the Xe-lamp used in the photocatalytic tests, an aliquot of 0.180 mL (V_2) was taken and 2 mL of a buffered solution of phenanthroline (0.015 M / 0.5 M H_2SO_4) were added together with distilled H_2O to give a final volume of 25 mL (V_3) which maintained in dark 90 minutes to ensure total complexes of Fe-Phenanthroline. The absorbance of the solution at 510 nm was recorded and the value was used to calculate the number of Fe^{2+} ions (nFe^{2+}) generated during the irradiation process through the following equation:

$$\mathbf{nFe^{2+} = V_1 \times V_3(A\text{–}A_0)/\ V_2 \times \varepsilon_0 \times 1000}$$

V_1 = volume of actinometer solution irradiated (mL)

V_2 = volume of aliquot taken for analysis (mL)

V_3 = final volume to which the aliquot V_2 is diluted (mL)

A = measured optical density at 510 nm

A_0 = measured optical density at 510 nm of a non-irradiated sample

ε_0 = experimental value of the molar extinction coefficient of the Fe^{2+} complex (11100 $L.mol^{-1}.cm^{-1}$).

The number of incident photons per second (# photons) is calculated using the following equation:

$$\textbf{Incident Photons } (s^{-1}) = (6.022\times10^{23}) \times nFe^{2}/\ \Phi_{\lambda}\times t$$

Φ_{λ} = quantum yield of Fe^{2+} formation (average 1.1) and t = time of irradiation (s).

The calculated incident photon per second per cm^2 after 1 and 10 minutes of $K_3Fe(C_2O_4)_3$ solution irradiation were found to be 3.87×10^{16} and 3.85×10^{16} s^{-1}, respectively.

CV and EIS experimental details

Cyclic voltammetry (CV) and electrochemical impedance spectroscopy (EIS) experiments were performed using a Biologic bipotentiostat (SP-300) in a three-electrode cell configuration. All measurements were carried out in 0.5 M Na_2SO_4 as a supporting electrolyte. CV measurements were performed at scan rates within the 20 –100 mV s-1 range (starting from high to low potential scan rate), in a home-made three-compartment glass cell. Glassy Carbon electrode of ca. 3 mm diameter was used as working electrode and a platinum wire as counter electrode. All potentials were recorded versus Ag/AgCl as a reference electrode. Prior to the CV measurements, the OCP of each electrode was monitored for at least 60 s. The peak potentials were evaluated from the first scan.

Working electrodes preparation

Glassy carbon electrodes were cleaned as follows: rinsed with DIW and methanol sequentially; polished consecutively with coarse diamond (1 µm) and fine alumina (0.05 µm) solution; soaked

in DIW and methanol sequentially while sonicating for 5 mins. 20 µL from 5 mg/mL MOFsolution was drop-casted on glassy carbon surface and left to dry at least 5 min.

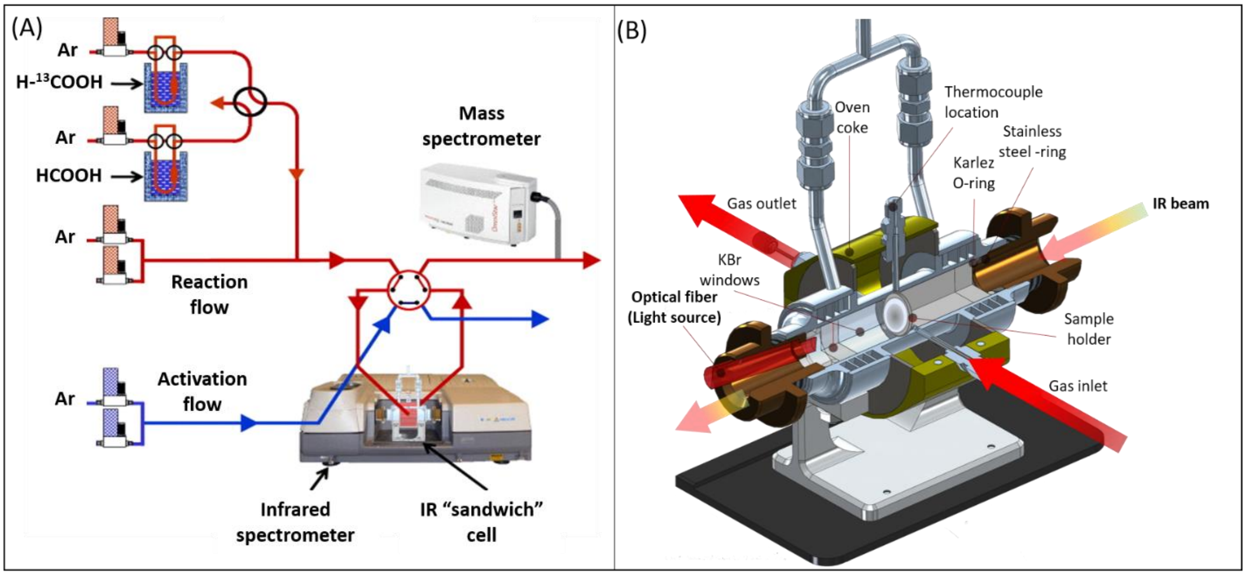

Scheme S1. (A) Operando setup and (B) Sandwich Reactor IR cell for studying Photodecomposition of FAc under visible light.

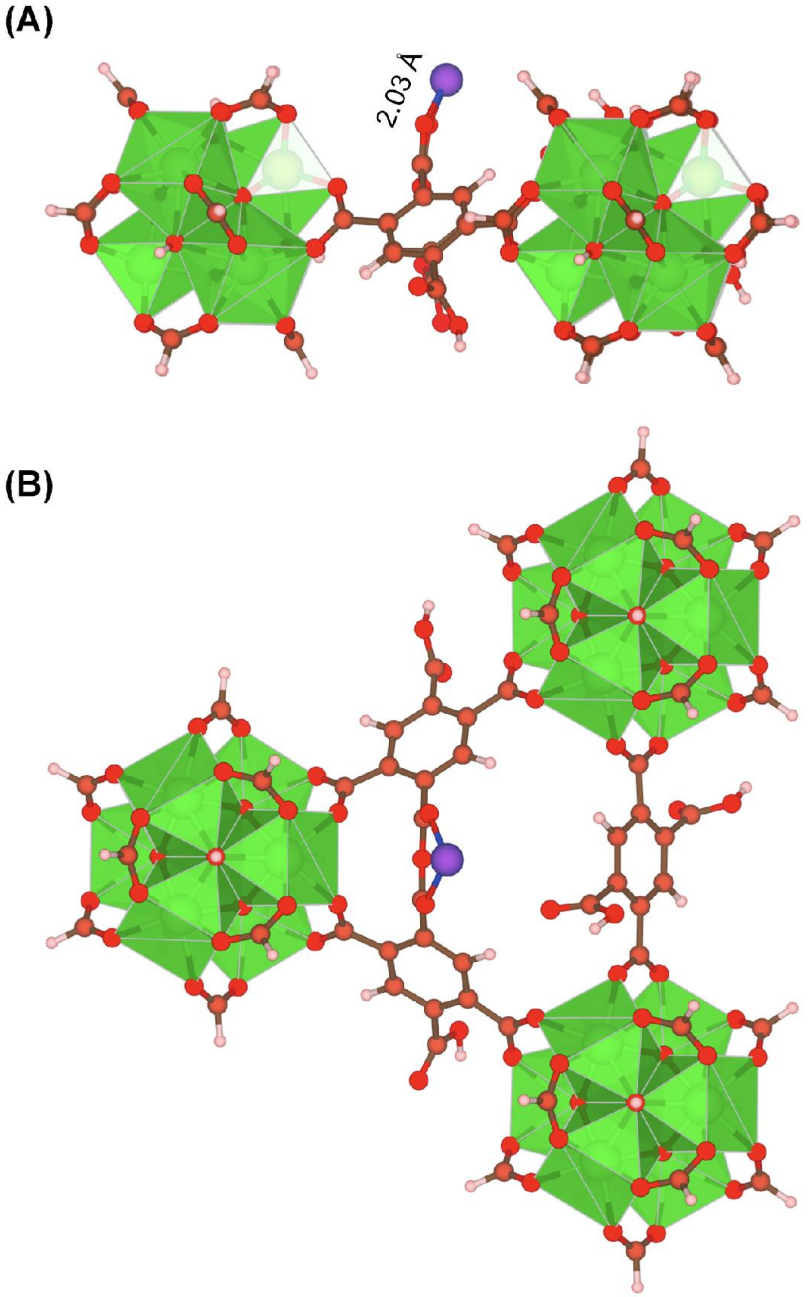

Figure S1. (a) Side and (b) top views of the DFT-optimized cluster model of UiO-66-$(COO)_2$-Cu. Color codes for the atoms: C, gray; Cu, blue; O, red; H, white; Zr, green.

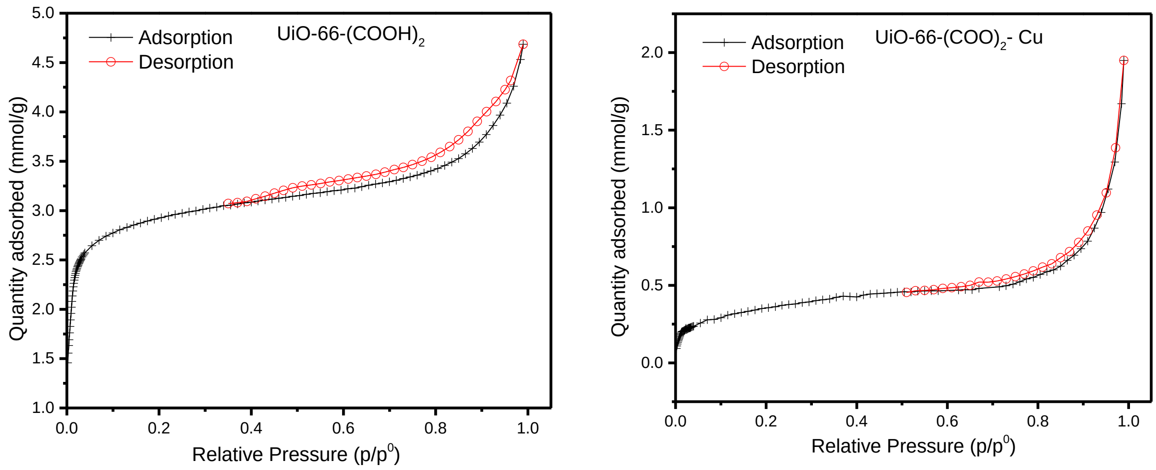

Figure S2. N_2 adsorption-desorption isotherms at 77 K for UiO-66-$(COOH)_2$ (left) and UiO-66-$(COO)_2$-Cu (right).

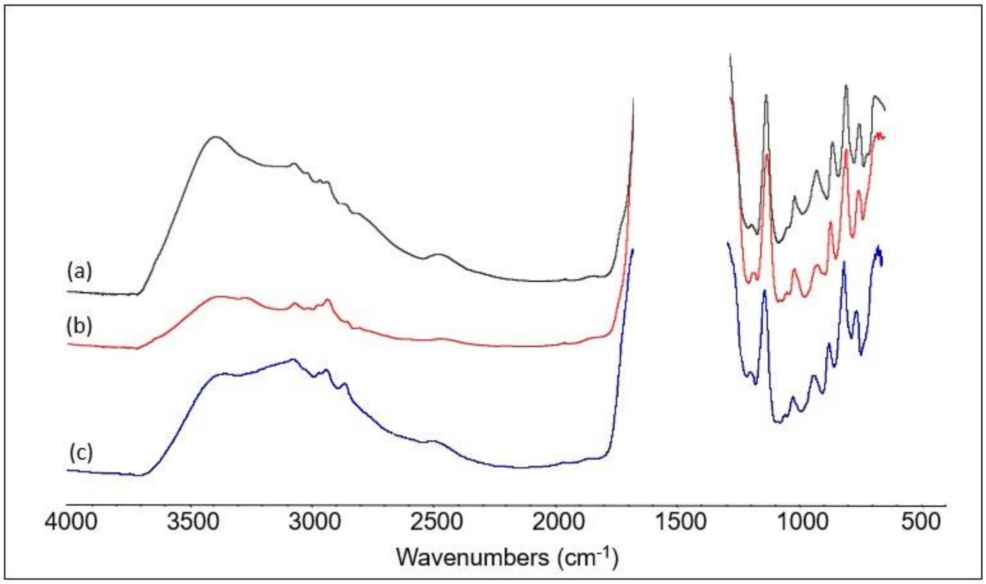

Figure S3. FTIR spectra of the UiO-66-$(COO)_2$-Cu before (a) and after (b) 20 minutes of activation under visible light irradiation under Ar flow (25 cc.min^{-1}). (c) corresponds to the FITR spectrum of the UiO-66-$(COO)_2$-Cu at the steady state after adsorption of 2400 ppm of FAc in Ar at 25°C with total flow of 25 cc.min^{-1}.

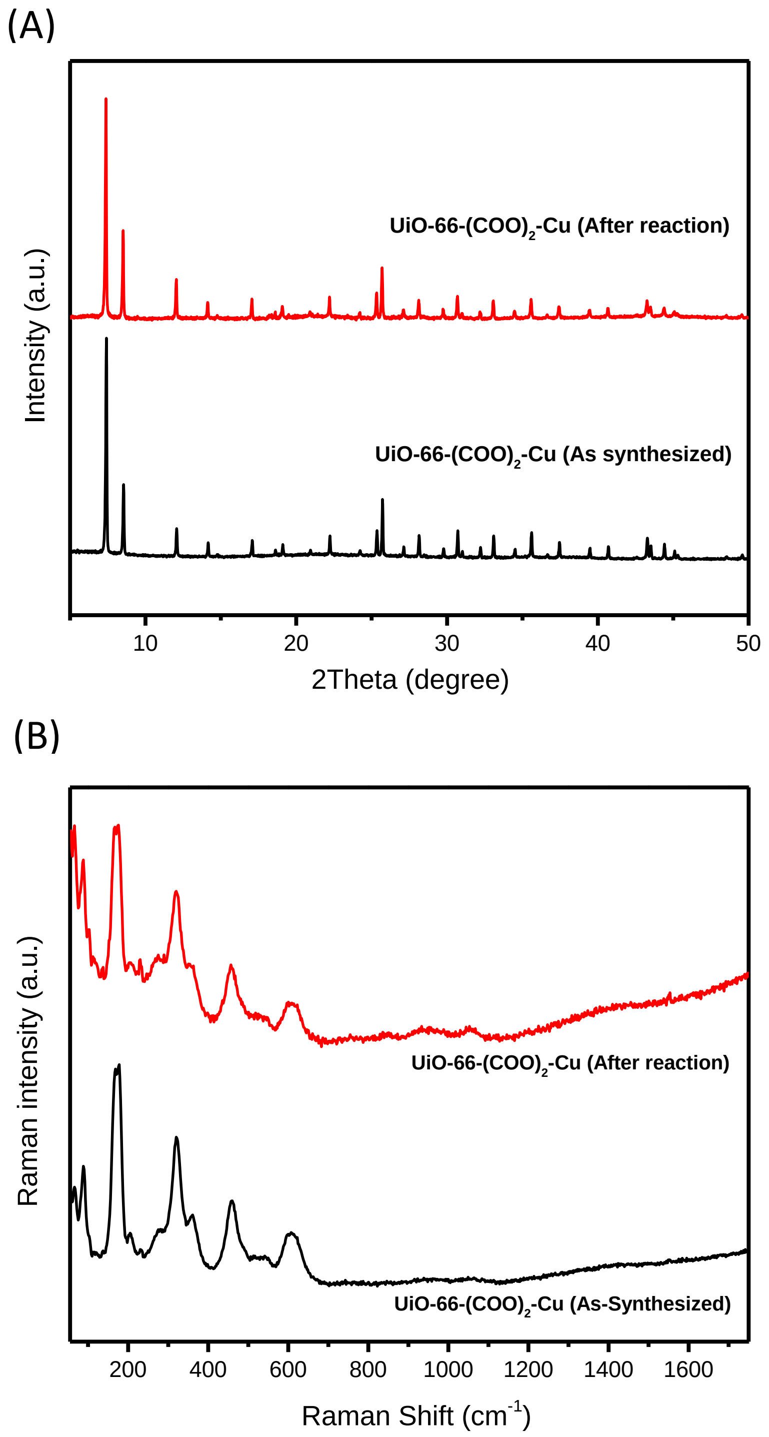

Figure S4. PXRD patterns (A) and Raman spectroscopy (B) of UiO-66-$(COO)_2$-Cu samples before and after reaction.

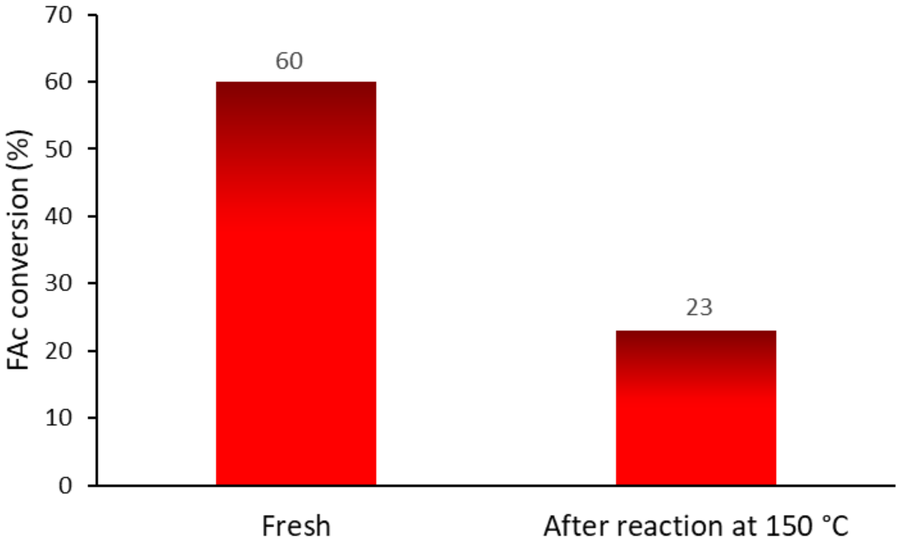

Figure S5. Formic acid conversion activity at room temperature of fresh UiO-66-$(COO)_2$-Cu and after reaction at 150 °C.

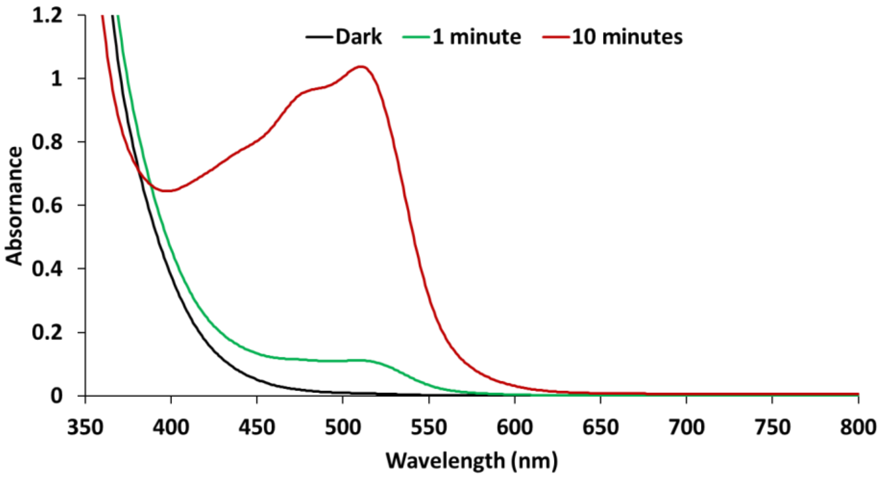

Figure S6. UV-Visible spectra of Fe-Phenanthroline solution (V_3) in dark, after 1 minutes and 10 minutes of irradiation.

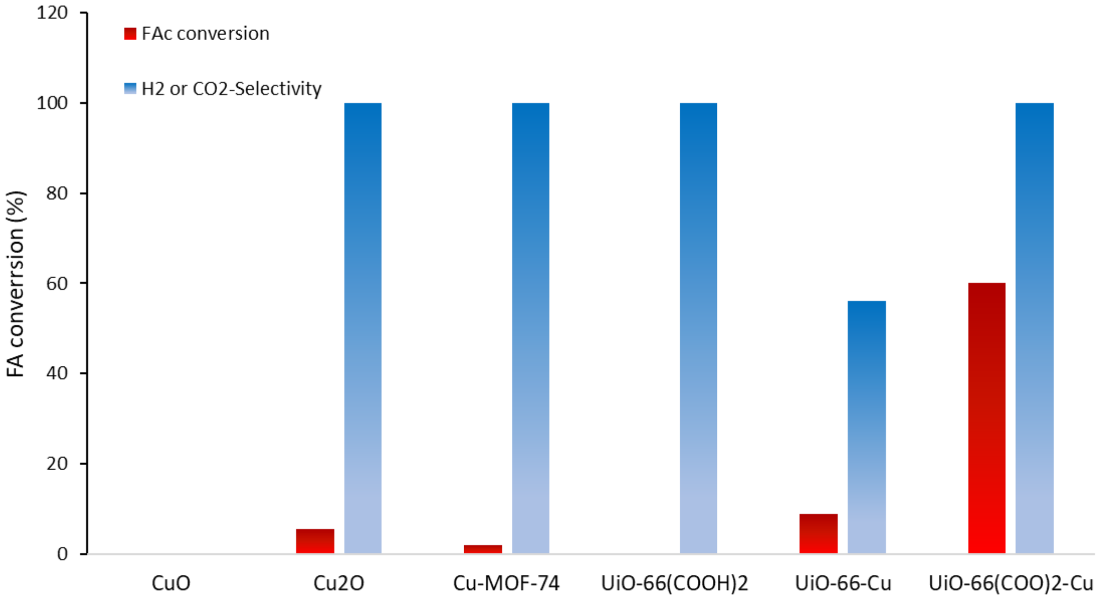

Figure S7. Formic acid conversion activity of CuO, Cu_2O, Cu-MOF-74, UiO-66-Cu and UiO-66-$(COOH)_2$ compared to our catalyst UiO-66-$(COO)_2$-Cu (18%) at room temperature.

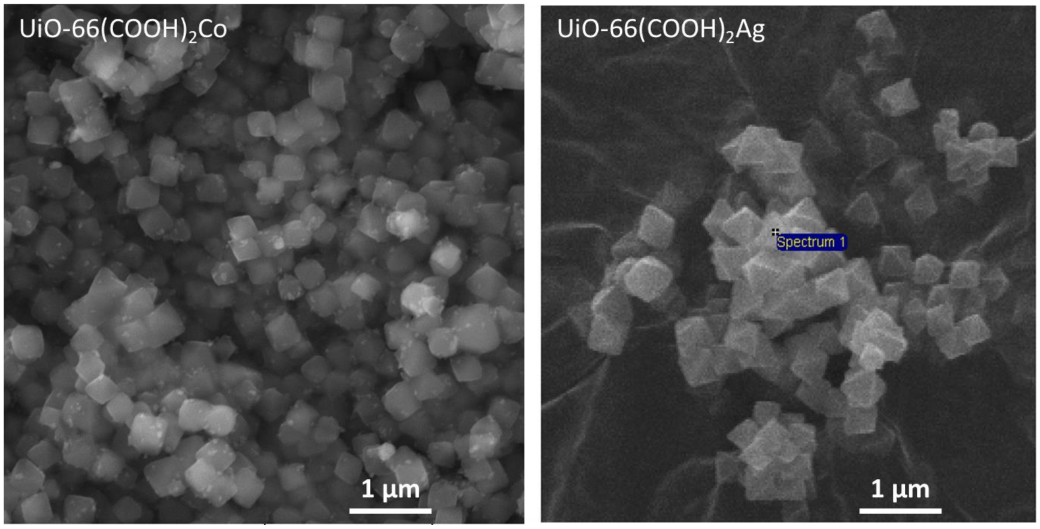

Element (Weight %)	UiO-66(COOH)$_2$Co	UiO-66(COOH)$_2$Ag
C	30.27	43.64
O	34.19	24.81
Zr	19.82	17.42
M (Co, Ag)	15.72	14.12

Figure S8. SEM images of UiO-66$(COOH)_2$Co and UiO-66$(COOH)_2$Ag along with the weight % of the various elements as determined by EDX.

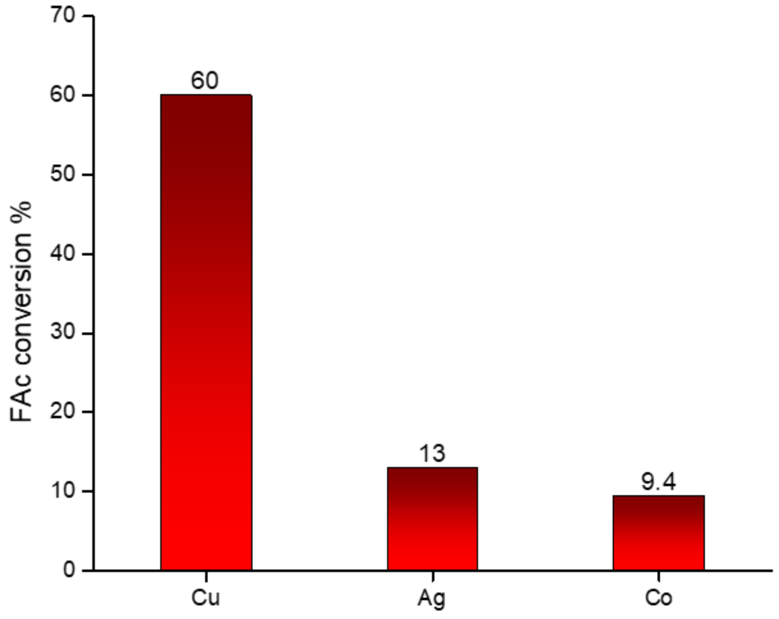

Figure S9. Formic acid conversion activity of UiO-66-$(COO)_2$-Cu, UiO-66-$(COO)_2$-Ag, and UiO-66-$(COO)_2$-Co at room temperature.

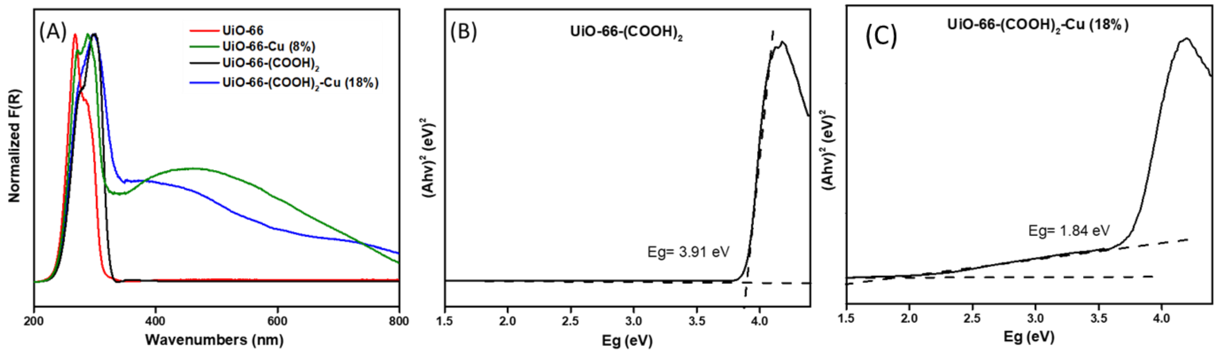

Figure S10. Absorbance spectra of UiO-66, UiO-66$(COOH)_2$ and their metalated forms (A) and tauc plot of UiO-66-$(COOH)_2$ (B), UiO-66-$(COOH)_2$-Cu (C).

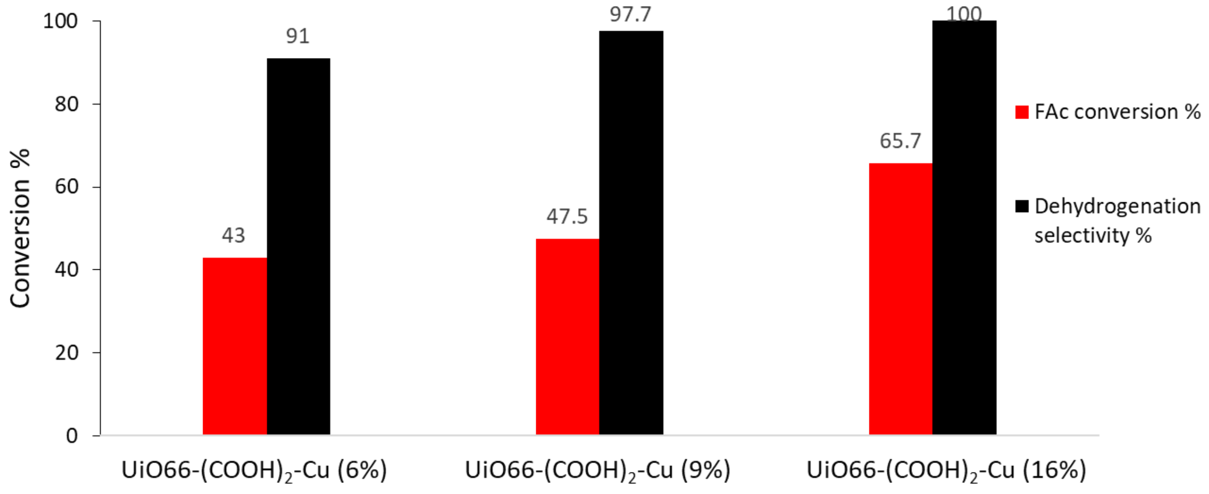

Figure S11. Formic acid conversion activity and dehydrogenation selectivity of UiO-66-$(COO)_2$-Cu with different Cu wt% loadings at room temperature.

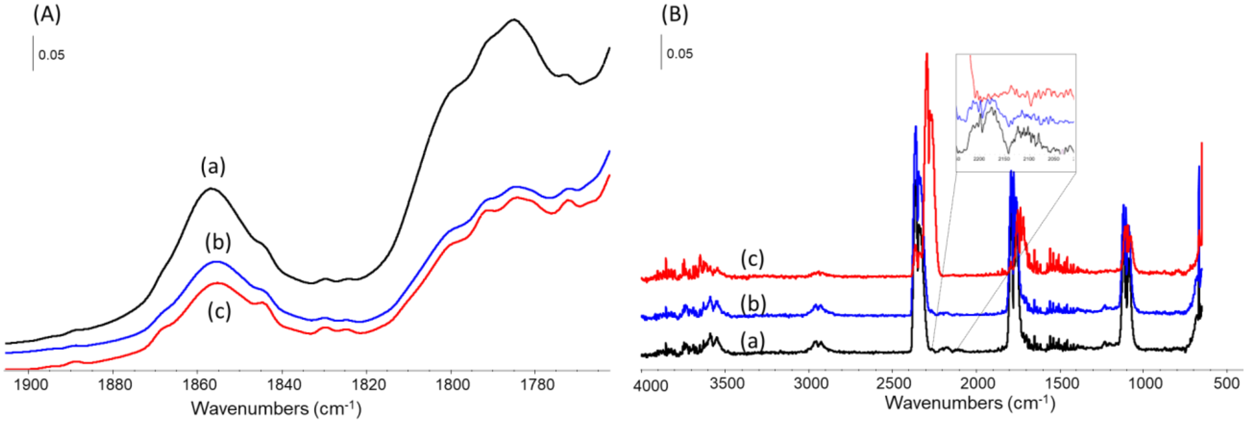

Figure S12. (A) FTIR surface spectra and (B) FTIR gas of (a) UiO-66-$(COOH)_2$-Cu (6%), (b) UiO-66-$(COOH)_2$-Cu (9%) and (c) UiO-66-$(COOH)_2$-Cu (16%) after 120 min of FAc photodecomposition under visible light. H-^{13}COOH is used in case of UiO-66-$(COOH)_2$-Cu (16%). CO peak can be detected for the 6 w% Cu and 9 w% Cu loaded samples.

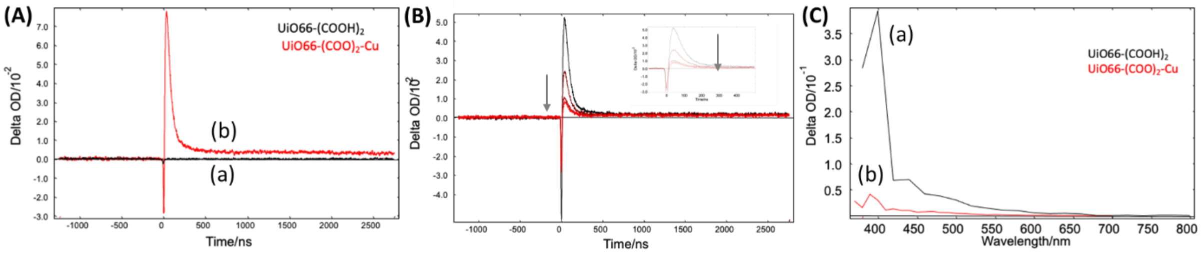

Figure S13. (A) Absorption transient (AT) decay at 450 nm of dispersed (a) UiO66-$(COOH)_2$ and (b)UiO66-$(COOH)_2$-Cu in water demonstrating the Cu-ligand charge transfer behavior in case of UiO66-$(COOH)_2$-Cu (no AT is observed for UiO66-$(COOH)_2$ sample). (B) Evolution of the Absorption transient decay of UiO66-$(COOH)_2$-Cu versus formic acid concentration in water (from top to bottom: 0; 0.9; 1.8; and 2.6 mM) demonstrating the leaching of the Cu at high FAc concentration. (C) PL spectra of (a) UiO66-$(COOH)_2$ and (b)UiO66-$(COOH)_2$-Cu demonstrating the highest photoluminescence behavior of UiO66-$(COOH)_2$. Excitation wavelength=532nm. Laser energy: 7mJ/cm^2."

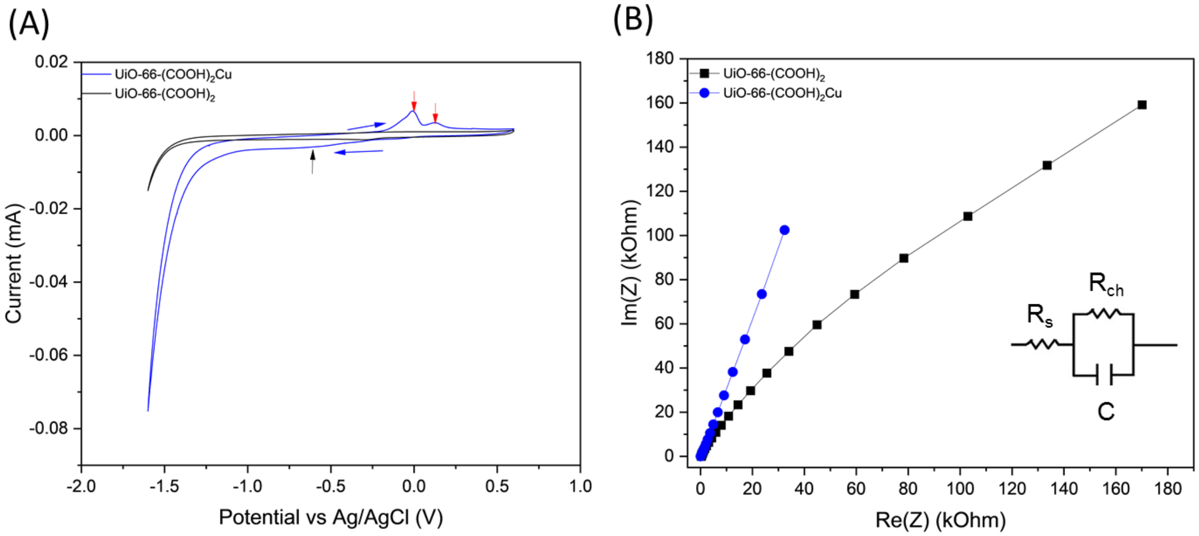

Figure S14. (a) Cyclic voltammograms of UiO-66$(COOH)_2$ (black) and its metalated form (blue) drop casted on a glassy carbon electrode in 0.5 M Na_2SO_4 solution (scan rate: 50 mVs^{-1}). Vertical arrows indicate the anodic and cathodic oxidation and reduction onset potentials while the horizontal ones show the potential sweep direction. (b) Nyquist plot in the frequency range (100 kHz to 100 mHz) of UiO-66$(COOH)_2$ (black) and its metalated form (blue) with inset

figure of the equivalent circuit where Rs is the solution resistance, Rch is the charge transfer resistance and C is a capacitor.

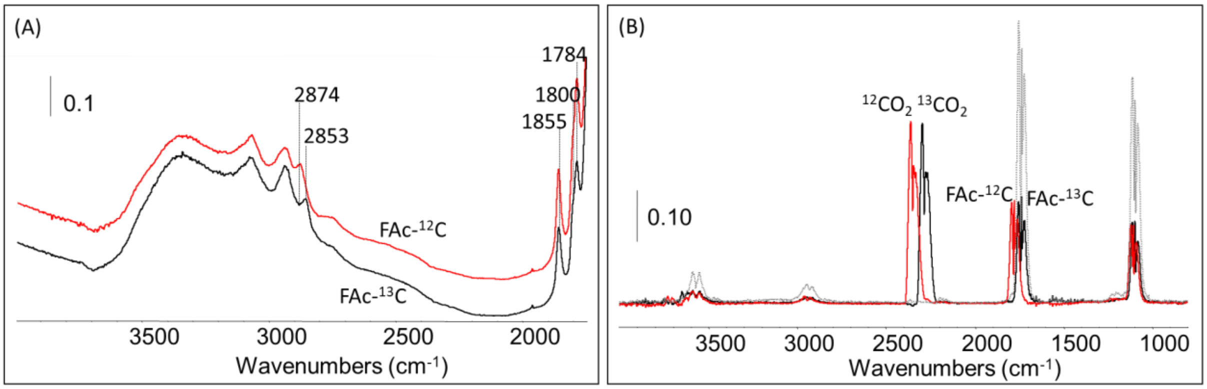

Figure S15. (A) FTIR spectra of UiO-66-$(COO)_2$-Cu surface and (B) FTIR the reaction gas phase at steady state during the reforming of unlabeled (FAc-^{12}C; red line) and labelled formic acid (FAc-^{13}C; black line) under visible-light irradiation. Dotted line corresponds to the FTIR spectra of the FAc-^{12}C before reaction. Reaction condition: total flow 25 cc/min; [FAc-^{12}C]=[FAc-^{13}C]=2400ppm (0.24%) in Ar; T=25°C; Xe-lamp 150 W with visible light pass filter (λ>390 nm); irradiance=71 mW/cm^2; m_{cat}=20 mg (self-supported pellet with surface of 1.6 cm^2).

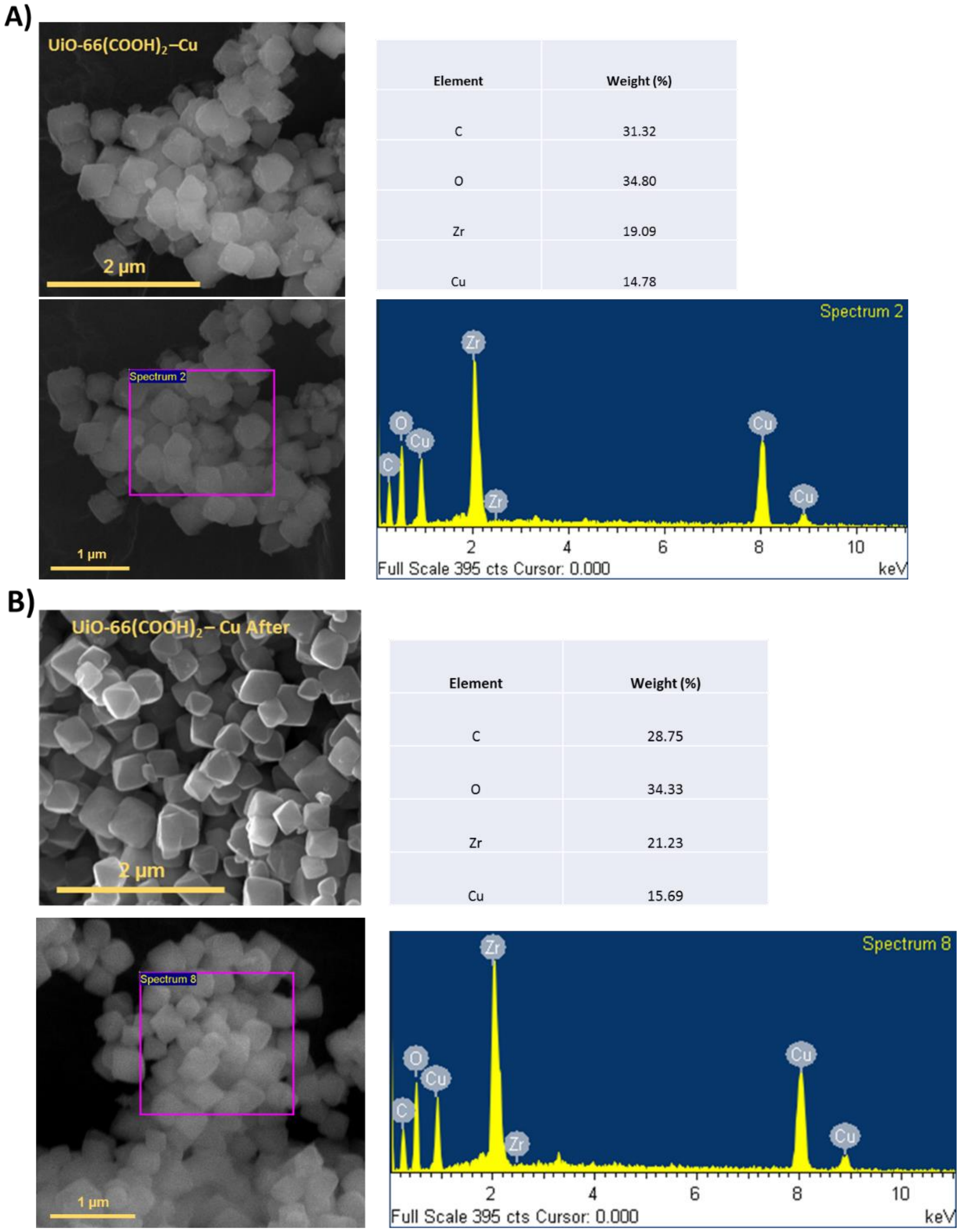

Figure S16. SEM images and EDX results of the (A) as-synthesized UiO-66-$(COO)_2$-Cu and (B) UiO-66-$(COO)_2$-Cu after reaction.

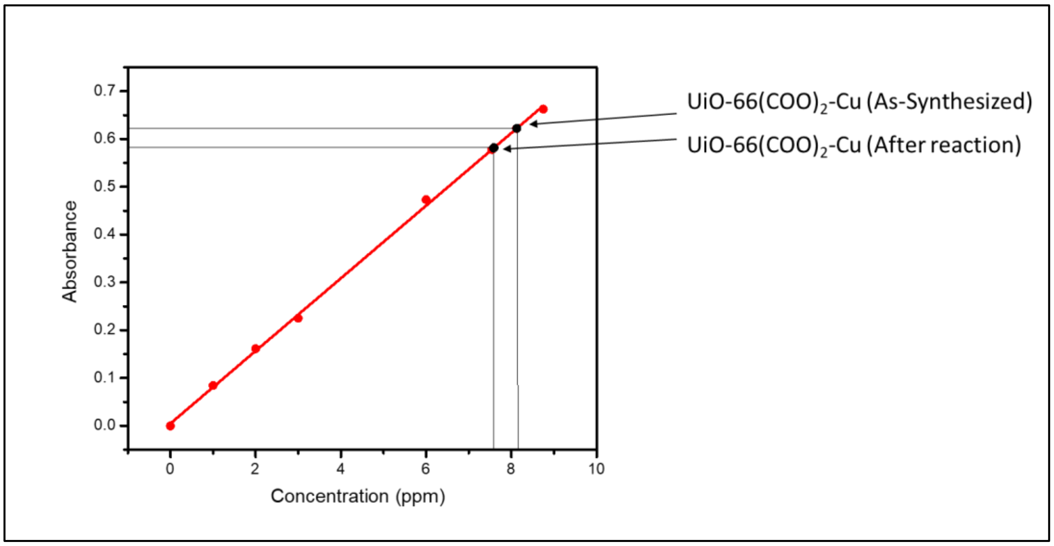

Sample	UiO-66(COO)$_2$-Cu (As-Synthesized)	UiO-66(COO)$_2$-Cu (After reaction)
mass of sample (mg)	0.438	0.418
Absorbance	0.62197	0.57811
Concentration (ppm)	8.1223	7.5448
% weight	19.28	18.77

Figure S17. Calibration curve of copper in flame atomic absorption spectrophotometer showing Cu % weight in UiO-66-(COO)$_2$-Cu before and after reaction.

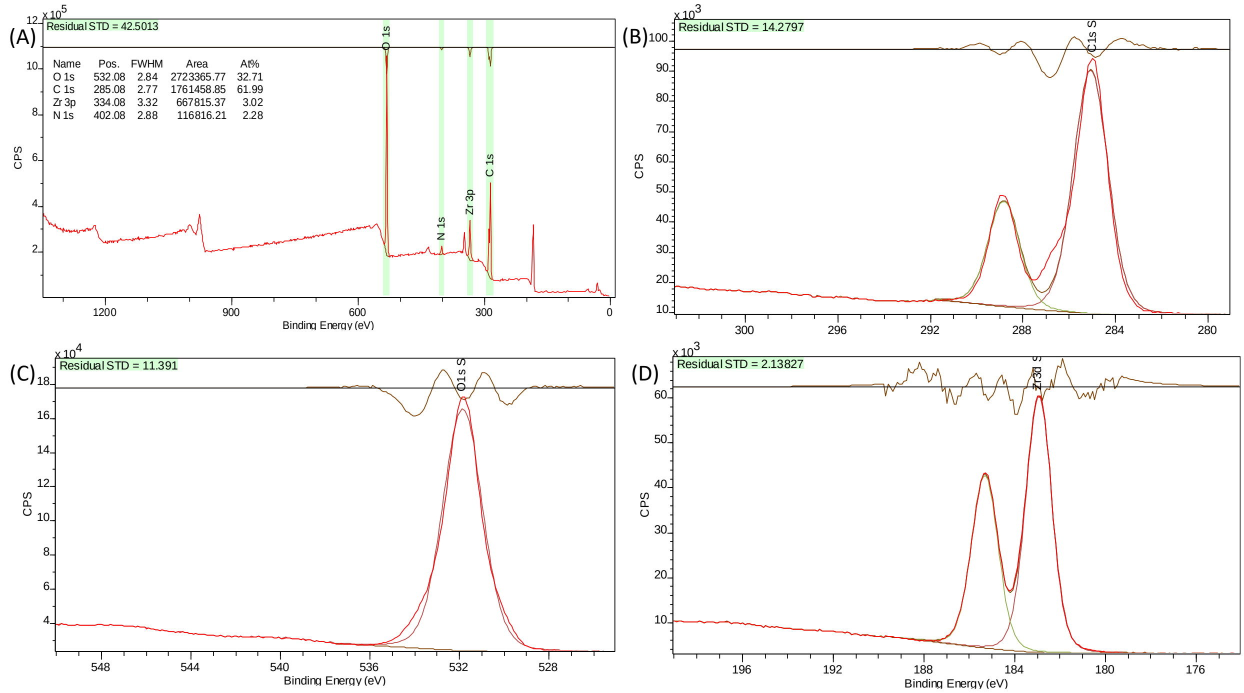

Figure S18. XPS Spectra of UiO-66(COOH)$_2$. (A) survey spectrum, (B) C 1s, (C)O 1s and (D) Zr 3d

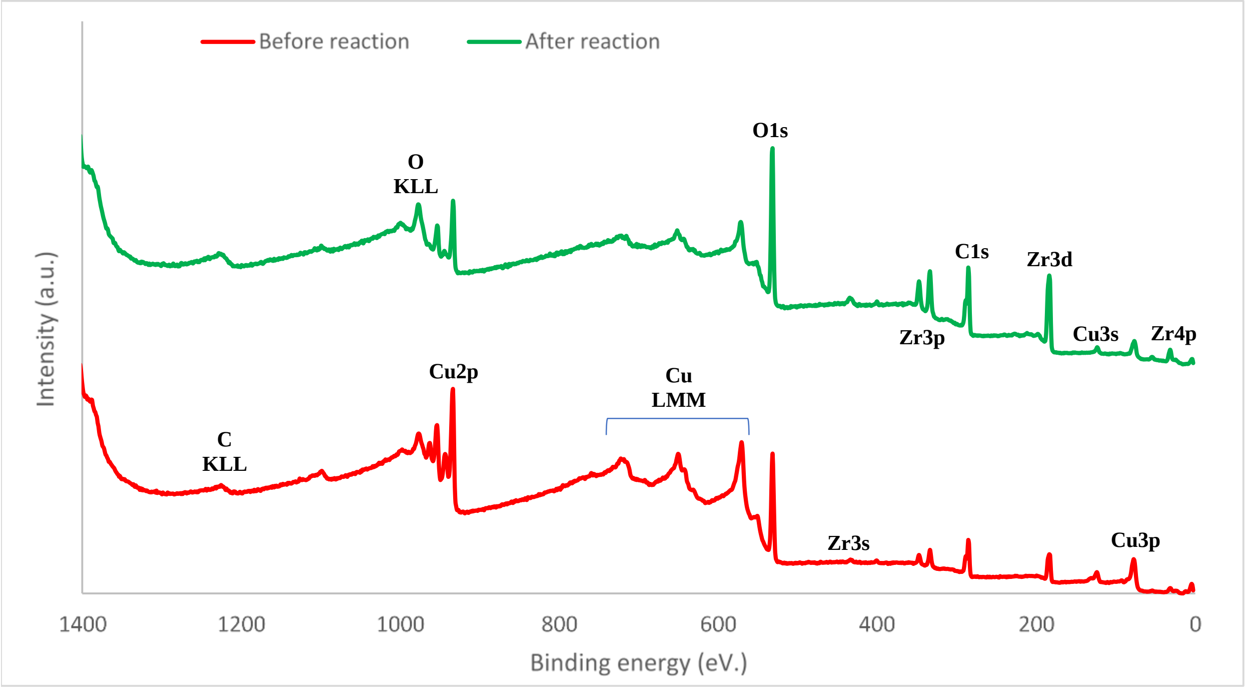

Figure S19. XPS spectra of UiO-66-(COO)$_2$-Cu before and after reaction.

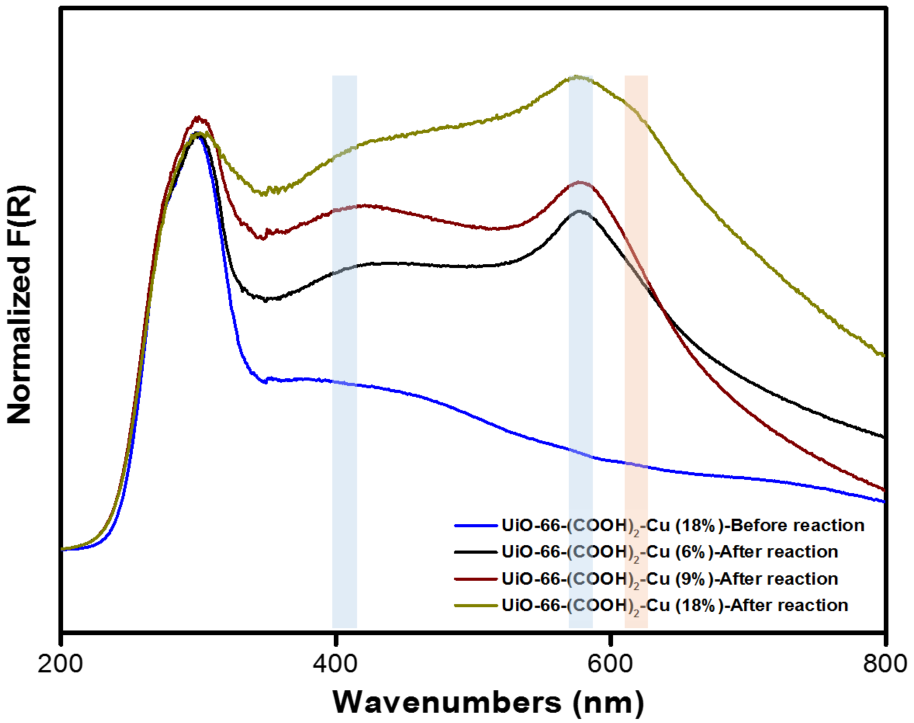

Figure S20. Diffuse reflectance spectra measured on the UiO-66$(COOH)_2$-Cu pellets before reaction compared to the samples of different Cu loading after reaction. The typical broad absorption band of Cu_2O in the visible region (420 and 485 nm) in addition to a shoulder peak evolved at 625 nm mainly in the highly loaded sample which corresponds to the plasmonic band of Cu are highlighted.

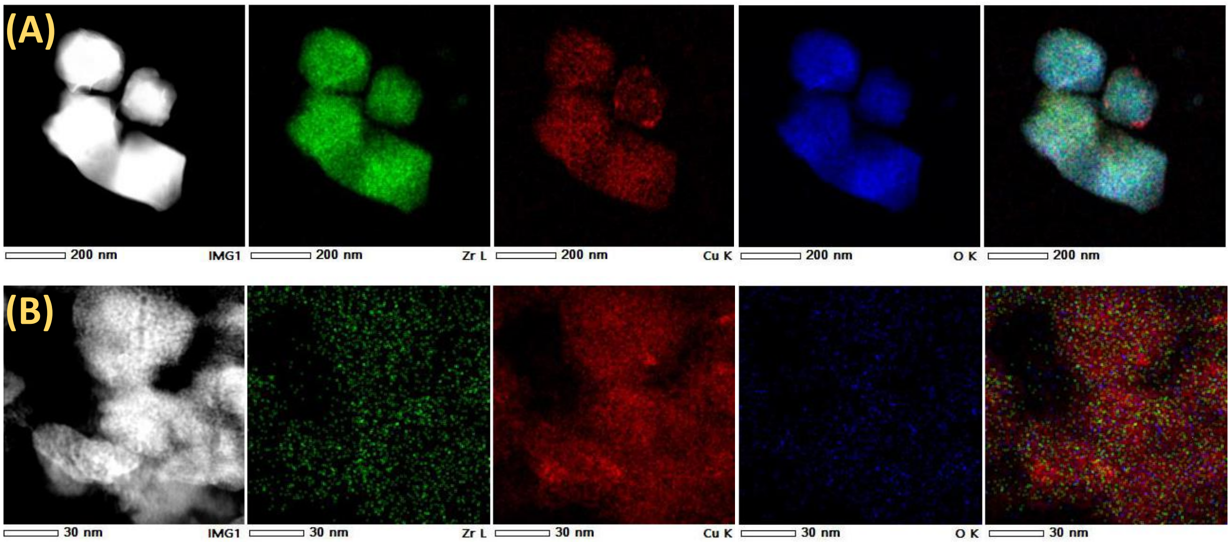

Figure S21. TEM images of UiO66-Cu (A: top) before and (B: bottom) after reaction with the corresponding EDX mapping showing the dispersion of Zr (green), Cu (red) and oxygen (blue) and their superposition.

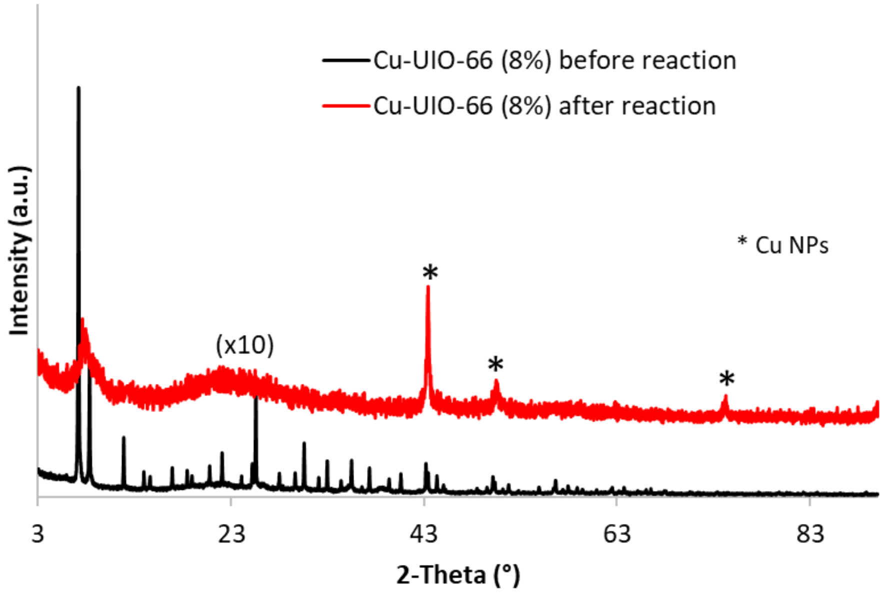

Figure S22. PXRD diffraction patterns of the copper metalated UiO-66 (8%) before and after reaction**.**

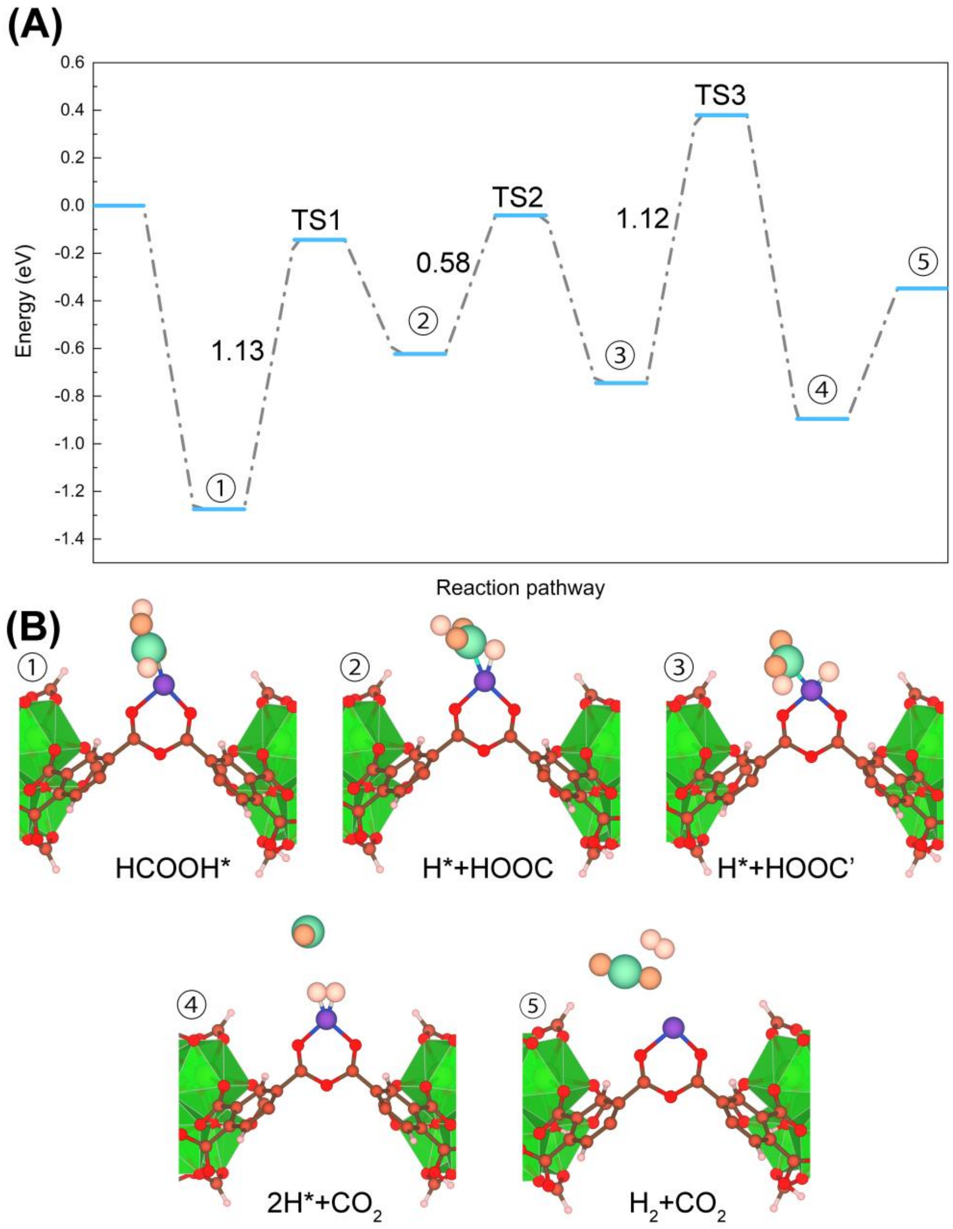

Figure S23. DFT-derived minimum energy COOH-dehydrogenation pathway by UiO-66-$(COO)_2$-Cu and the (b) corresponding illustrative snapshots of the different intermediate species. The energy barriers (expressed in eV) for the 3 transition states (TS) are also shown in the figure. Color codes for the MOF: C, gray; Cu, blue; O, red; H, white; Zr, green. Color codes for the adsorbed molecules: C, light blue; O, orange; H, white. The total free energy of UiO-66-$(COO)_2$)-Cu structure with gas-phase HCO_2H molecule is set as the zero in the Gibbs free energy profile.

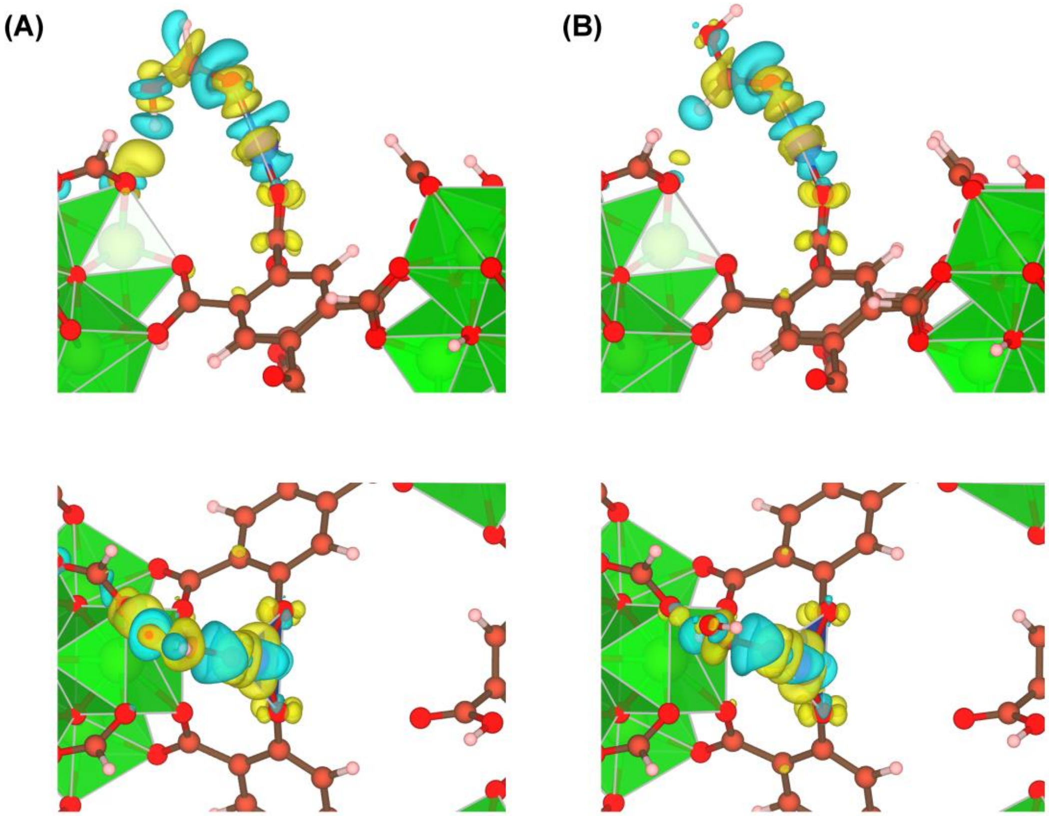

Figure S24. Side (upper) and top (lower) views of the charge density difference between the chemisorbed HCOOH and UiO-66-$(COO)_2$-Cu MOF structure followed by (a) HCOO-dehydrogenation and (b) COOH-dehydrogenation routes, respectively. The isosurface value was set to be 0.02. Green and blue refer to electron accumulation and depletion regions. Color codes for the MOF: C, gray; Cu, blue; O, red; H, white; Zr, green. Color codes for the adsorbed molecules: C, light blue; O, orange; H, white.

Table S1. FAc conversion (%), conversion yield (mmol.g^{-1}.cm^{-2}) and the reaction selectivity over CuO, Cu_2O, UiO-66$(COOH)_2$, UiO-66-Cu and UiO-66-$(COO)_2$-Cu. Reaction condition: total flow 25 cc/min; [FAc]=2400ppm (0.24%) in Ar; T=25°C; Xe-lamp 150 W with visible light pass filter (λ>390 nm); irradiance=71 mW/cm^2; mcat=20 mg (self-supported pellet with surface of 1.6 cm^2).

Samples	Conversion %	Conversion yield (mmol.g^{-1}.cm^{-2})	CO_2 selectivity (%)	CO selectivity (%)
CuO	0.07	0.00	n.d	n.d
Cu_2O	5.6	5.53	>99	-
Cu-MOF-74	2.0	1.60	100	-
UiO-66-$(COOH)_2$	0	0.00	-	-
UiO-66-Cu	8.8	7.15	60	40
UiO-66-$(COO)_2$-Cu	60	48.78	100	0
UiO-66-$(COO)_2$-Ag	13.0	10.46	100	0
UiO-66-$(COO)_2$-Co	9.4	7.56	100	0

[a]error on the conversion % is ± 9%

Table S2. Conversion rates for FAc dehydrogenation to H_2 using different homogeneous and heterogeneous photocatalysts under different reaction conditions.

Type	Photocatalyst	Light source/Temperature	Phase	H_2 production rate in mmol/g./h (in mmol/g/cm^2)	Apparent quantum yield (%)	Dehydrogenation selectivity %	Stability test	Remarque	Ref.
Homogeneous	Fe_3CO_{12}/PPh_3/tpy	Xe lamp (300W > 385 nm) T = 80 °C	Liquid	2.7	-	Traces of CO	stable >24 h	Relatively High temperature	4
	[Cp*Ir(bpy-OMe)(Cl)]$^+$	LED lamp (λ = 460 nm), RT	Liquid	26.7	$\Phi_{406\ nm}$ = 0.05 $\Phi_{503\ nm}$ = 0.10	>95	deactivation after 72 h of illumination	Ir-based catalyst	5
	Dinuclear Ir complex	LED lamp (3W, λ = 395 nm), RT	Liquid	0.35	-	-	stable after continuous switching cycles for 54 h	Ir-based catalyst	6
	$[RuCl_2(C_6H_6)]_2$ + 12PPh	Xe lamp (300 W, > 380 nm), T = 45 °C	Liquid	120	-	-	stable for >300 min	Ru-based catalyst	7
	CdS/Fe-salen	Xe lamp (300W, > 420 nm), RT	Liquid	150	$\Phi_{420\ nm}$ = 16.8	67	good stability during 30 hours	Low stability of CdS in acidic media!	8
Heterogenous	CuO+TiO_2	solar light, RT	Liquid	2.4	-	-	Decrease of activity after 5 cycles (24h/cycle) due to Cu leaching	Low activity and no information on the selectivity	9
	Pt/Cu_2O	Hg lamp (280 mW/cm^2 >420 nm), RT	Liquid	0.1	-	100	stable for 60 h	UV, Pt-based catalyst	10
	CdS-ZnS/Ru	Xenon lamp (300W, 70 mW/cm^2 >420 nm), RT	Liquid	5.8	$\Phi_{visible\ light}$ =20	-	Stable for 10 cycles (4h/cycle)	Ru-based catalyst, Low stability of CdS in acidic media!	11
	Pt-CdS	Hg lamp (400 W> 420 nm), RT	Liquid	1.2	$\Phi_{420\ nm}$ = 21.4	-	stable for 5 cycles (5h/cycle)	UV, Pt-based catalyst, low stability of CdS in acidic media!	12
	FeP@CdS	Xe lamp (300W > 420 nm), RT	Liquid	278	$\Phi_{420\ nm}$ = 54	100	stable for 6 cycles (4h/cycle)		13
	PdAg@C_3N_4	Xe lamp (300W > 400 nm), RT	Liquid	3.9	-	100	stable for 4 cycles (1h/cycle)	Pd/Ag-based catalyst	14
	Au Pd/C_3N_4	Xe lamp (300W > 400 nm), RT	Liquid	3.57	-	100	stable for 4 cycles (20h/cycle)	Au/Pd-based catalyst	15

	TiO_2@NiPc@GO	Xe lamp (300W > 420 nm), T = 55 °C	Liquid	15.3	-	100	stable for 5 cycles (24h/cycle)	T=55°C	16
	CdS@ZIF-8	Xe lamp (300 W> 420 nm), RT	Liquid	0.9	-	88	-	No information on the stability	17
	Co-sal-NH_2-MIL-68@In_2S_3	Xe lamp (300 W> 420 nm), RT	Liquid	18.7	$\Phi_{420\ nm}$ = 3.8	99.9	stable for 3 cycles (24h/cycle)	In-based photocatalyst, low activity	18
	Au@Pd/MOF (UiO-66($Zr_{85}Ti_{15}$)	Xe lamp (500 W, 320 mW/cm^2, > 420 nm), RT	Liquid	5.4	-	100	slight decrease of activity after 3 cycles (1h/cycle)	Au/Pd-based catalyst,	19
	Ag^0@ZX-V	Xe lamp (200W, > 400 nm), RT	Liquid	2	-	>99	stable for 2 cycles (5h/cycle)		
	UiO-66-(COO_2)-Cu	Xe lamp (150W, 71 mW/cm^2, > 390 nm), RT	gas under continuous flow	4.8 (50)	$\Phi_{visible\ light}$ =10.6	>99.9	stable for 3 cycles (24h/cycle)		**this work**

References:

[1] Mortada, B.; Matar, T. A.; Sakaya, A.; Atallah, H.; Kara Ali, Z.; Karam, P.; Hmadeh, M., Postmetalated Zirconium Metal Organic Frameworks as a Highly Potent Bactericide. *Inorganic Chemistry* **2017,** *56* (8), 4739-4744.

[2] Jrad, A.; Hmadeh, M.; Abu Tarboush, B. J.; Awada, G.; Ahmad, M., Structural engineering of Zr-based metal-organic framework catalysts for optimized biofuel additives production. *Chemical Engineering Journal* **2020,** *382*, 122793.

[3] Sanz, R.; Martínez, F.; Orcajo, G.; Wojtas, L.; Briones, D., Synthesis of a honeycomb-like Cu-based metal–organic framework and its carbon dioxide adsorption behaviour. *Dalton Transactions* **2013,** *42* (7), 2392-2398.

[4] Boddien, A.; Loges, B.; Gärtner, F.; Torborg, C.; Fumino, K.; Junge, H.; Ludwig, R.; Beller, M., Iron-Catalyzed Hydrogen Production from Formic Acid. *Journal of the American Chemical Society* **2010,** *132* (26), 8924-8934.

[5] Barrett, S. M.; Slattery, S. A.; Miller, A. J. M., Photochemical Formic Acid Dehydrogenation by Iridium Complexes: Understanding Mechanism and Overcoming Deactivation. *ACS Catalysis* **2015,** *5* (11), 6320-6327.

[6] Sofue, Y.; Nomura, K.; Inagaki, A., On-demand hydrogen production from formic acid by light-active dinuclear iridium catalysts. *Chemical Communications* **2020,** *56* (33), 4519-4522.

[7] Loges, B.; Boddien, A.; Junge, H.; Noyes, J. R.; Baumann, W.; Beller, M., Hydrogen generation: catalytic acceleration and control by light. *Chemical Communications* **2009,** (28), 4185-4187.

[8] Irfan, R. M.; Wang, T.; Jiang, D.; Yue, Q.; Zhang, L.; Cao, H.; Pan, Y.; Du, P., Homogeneous Molecular Iron Catalysts for Direct Photocatalytic Conversion of Formic Acid to Syngas (CO+H2). *Angewandte Chemie International Edition* **2020,** *59* (35), 14818-14824.

[9] Maldonado, M. I., Saggioro, E., Peral, J., Rodríguez-Castellón, E., Jiménez-Jiménez, J., & Malato, S. Hydrogen generation by irradiation of commercial CuO+ TiO_2 mixtures at solar pilot plant scale and in presence of organic electron donors. *Applied Catalysis B: Environmental,* **2019**, *257*, 117890.

[10] Kakuta, S., & Abe, T. A novel example of molecular hydrogen generation from formic acid at visible-light-responsive photocatalyst. *Acs Applied Materials & Interfaces* **2009**, *1*(12), 2707-2710.

[11] Wang, X., Peng, W. C., & Li, X. Y**.** Photocatalytic hydrogen generation with simultaneous organic degradation by composite CdS–ZnS nanoparticles under visible light. *International journal of hydrogen energy* **2014**, 39(25), 13454-13461.

[12] Li, Y.; Tang, L.; Peng, S.; Li, Z.; Lu, G., Phosphate-assisted hydrothermal synthesis of hexagonal CdS for efficient photocatalytic hydrogen evolution. *CrystEngComm* **2012,** *14* (20), 6974-6982.

[13] Wang, T.; Yang, L.; Jiang, D.; Cao, H.; Minja, A. C.; Du, P., CdS Nanorods Anchored with Crystalline FeP Nanoparticles for Efficient Photocatalytic Formic Acid Dehydrogenation. *ACS Applied Materials & Interfaces* **2021,** *13* (20), 23751-23759.

[14] Liu, H., Liu, X., Yang, W., Shen, M., Geng, S., Yu, C., ... & Yu, Y. Photocatalytic dehydrogenation of formic acid promoted by a superior PdAg@ gC_3N_4 Mott–Schottky heterojunction. *Journal of Materials Chemistry A* **2019**, *7*(5), 2022-2026.

[15] Zhang, S., Li, M., Zhao, J., Wang, H., Zhu, X., Han, J., & Liu, X. Plasmonic AuPd-based Mott-Schottky photocatalyst for synergistically enhanced hydrogen evolution from formic acid and aldehyde. *Applied Catalysis B: Environmental* **2019**, *252*, 24-32.

[16] Keshipour, S.; Mohammad-Alizadeh, S., Nickel phthalocyanine@graphene oxide/TiO_2 as an efficient degradation catalyst of formic acid toward hydrogen production. *Scientific Reports* **2021,** *11* (1), 16148.

[17] Zeng, M., Chai, Z., Deng, X., Li, Q., Feng, S., Wang, J., & Xu, D. Core–shell CdS@ ZIF-8 structures for improved selectivity in photocatalytic H 2 generation from formic acid. *Nano Research* **2016**, 9(9), 2729-2734.

[18] Zhang, M.; Lin, W.; Ma, L.; Pi, Y.; Wang, T., An in situ derived MOF@In2S3 heterojunction stabilizes Co(ii)-salicylaldimine for efficient photocatalytic formic acid dehydrogenation. *Chemical Communications* **2022**.

[19] Wen, M., Mori, K., Kuwahara, Y., & Yamashita, H. Plasmonic Au@ Pd nanoparticles supported on a basic metal–organic framework: synergic boosting of H_2 production from formic acid. *ACS Energy Letters* **2017**, *2*(1), 1-7.
